# Antiproliferative Activity of Pt(IV) Conjugates Containing the Non-Steroidal Anti-Inflammatory Drugs (NSAIDs) Ketoprofen and Naproxen †

**DOI:** 10.3390/ijms20123074

**Published:** 2019-06-24

**Authors:** Mauro Ravera, Ilaria Zanellato, Elisabetta Gabano, Elena Perin, Beatrice Rangone, Marco Coppola, Domenico Osella

**Affiliations:** Dipartimento di Scienze e Innovazione Tecnologica, Università del Piemonte Orientale, Viale T. Michel 11, 15121 Alessandria, Italy; mauro.ravera@uniupo.it (M.R.); ilaria.zanellato@uniupo.it (I.Z.); elisabetta.gabano@uniupo.it (E.G.); elena.perin@uniupo.it (E.P.); beatrice.rangone@uniupo.it (B.R.); marco.coppola@uniupo.it (M.C.)

**Keywords:** multifunctional Pt(IV) prodrugs, antiproliferative activity, NSAID, COX-2, NAG-1, mesothelioma

## Abstract

Cisplatin and several non-steroidal anti-inflammatory drugs (NSAIDs) have been proven to act synergistically or at least additively on several tumor cell lines. Dual-action cisplatin-based Pt(IV) combos containing ketoprofen and naproxen offer good antiproliferative performance on a panel of human tumor cell lines, including a malignant pleural mesothelioma (MPM) one, a very chemoresistant tumor. The main reason of the increased activity relies on the enhanced lipophilicity of these Pt(IV) conjugates that in turn promotes increased cellular accumulation. A quick Pt(IV)→Pt(II) reduction generates the active cisplatin metabolite. The NSAID adjuvant action seems to be almost independent from cyclooxygenase-2 (*COX-2*) expression in the tumor cells under investigation (lung A-549, colon HT-29, HCT 116, SW480, ovarian A2780, and biphasic MPM MSTO-211H), but it seems to rely (at least in part) on the activation of the NSAID activated gene, *NAG-1* (a member of the transforming growth factor beta, *TGF-β*, superfamily), which has been suggested to be involved in NSAID antiproliferative activity.

## 1. Introduction

There are two main problems when a single drug is used in clinics: (i) There is no pharmacological agent with a “perfect” selectivity (and this causes systemic toxicity); (ii) even the most successful therapies may lose potency with time because of the development of acquired resistance. Combination therapy is aimed to overcome these limitations: Employing two or more synergistic drugs together reduces their individual dose and therefore their toxicity. At the same time, when two or more drugs which have different biochemical mechanisms of action are combined, the probability of selecting tumor cells resistant to all the drugs is reduced.

These considerations apply to Pt-based anticancer compounds, the prototype of which is cisplatin ((*SP*-4-2)-diamminedichloridoplatinum(II) and cis-[PtCl_2_(NH_3_)_2_]) [1]. The development of a cisplatin-based combination chemotherapy such as the PVB (cisplatin, vinblastine and bleomycin) protocol in the late 1970s resulted in a dramatic improvement in the prognosis of patients with metastatic germ cell tumors, and overall cure rates exceeded 80%. Today, the bleomycin, etoposide, cisplatin (BEP) combination is considered to be the standard treatment of patients with testicular cancer, with survival rates of 90% [2].

However, the administration of two or more drugs can suffer from drawbacks such as different bioavailabilities, the pharmacokinetics and metabolism of the drugs, and possible drug–drug interactions. The preparation of a single molecule containing different chemically bonded drugs (combo) may represent a more effective strategy to address this task [3].

**Figure 1:**
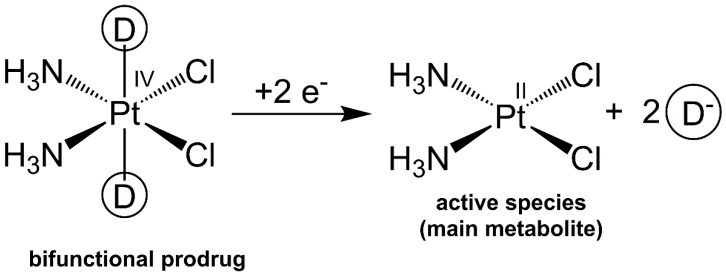
Reduction reaction of a cisplatin-based Pt(IV) prodrug; D = second drug.

In this framework, octahedral Pt(IV) complexes play a role because of their peculiar chemical features [4,5,6,7]. The octahedral, coordinatively saturated geometry of Pt(IV) is generally characterized by kinetic inertness that can minimize off-target reactions and allow for oral administration. Additionally, Pt(IV) complexes are selectively (or at least preferentially) reduced in the hypoxic tumor environment to the corresponding cytotoxic Pt(II) metabolites with the loss of their axial ligands (activation by reduction) [6,8,9,10]. Recently, Pt(IV) chemistry has been used to design multi-functional conjugates. One or two adjuvant or synergistic agents are conjugated to the octahedral Pt(IV) core in axial position so that they and the parent Pt(II) drug will be released simultaneously and will act in a synergistic or at least in an additive way [4,7,11,12,13,14] (Figure 1).

Various agents with different cellular targets have been coordinated to Pt(IV) complexes to obtain such combos, and a number of them contain the inhibitors of cyclooxygenase (COX) enzymes. There are two main isoforms of COX, namely COX-1 and COX-2, responsible for the formation of biological mediators of inflammation, including prostaglandins (as prostaglandin E_2_, PGE_2_), prostacyclin, and thromboxane. *COX-1* is constitutively expressed in most tissues, whereas the COX-2 level is generally very low unless induced in response to inflammatory and other physiological stimuli. Indeed, an enhanced *COX-2* expression is found in several tumors, and it appears to play a role in carcinogenesis [15]. For this reason, COX inhibitors, including the large class of non-steroidal anti-inflammatory drugs (NSAIDs), as well as the COX-2 selective inhibitors (e.g., celecoxib), are suggested for cancer prevention, especially as colon adenocarcinoma is concerned [16]. NSAIDs are commonly used for their analgesic and antipyretic effects by blocking the generation of PGE_2_ via the inhibition of COX activity [17,18,19]. Regrettably, NSAIDs showed a modest tumor growth inhibition when employed as a single agent; however, they have been tested as adjuvant agents in some chemotherapeutic protocols [20]. The overall mechanisms of their action are so far not fully understood, but both COX-dependent and -independent pathways seem to play a role [17,18].

Conventional NSAIDs, such as aspirin, ibuprofen, naproxen, indomethacin, and several others, inhibit both COX-1 and COX-2. Unfortunately, NSAIDs seem to exercise their anti-cancer adjuvant effects only through the inhibition of the inducible COX-2, whereas unwanted side effects, such as gastrointestinal problems, are thought to arise from the inhibition of the constitutive COX-1 [21,22,23].

**Figure 2:**
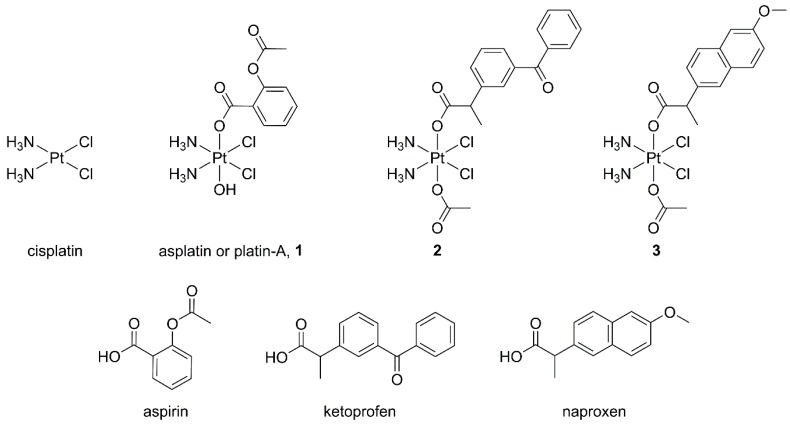
Sketch of the compounds under investigation.

The first example of a Pt(IV) complex containing acetylsalicylic acid (2-acetoxybenzoic acid, aspirin) as a ligand is asplatin or platin-A (**1**, Figure 2). Asplatin exhibited an antiproliferative activity comparable or slightly better to that of cisplatin alone or of an equimolar mixture of cisplatin and aspirin [24,25,26]. Interestingly, an in vivo assay has demonstrated that asplatin possesses a higher antitumor efficacy with lower toxicity in comparison with cisplatin [25].

Following this research, several cisplatin-, oxaliplatin-, or kiteplatin-based Pt(IV) complexes containing NSAIDs (i.e., indomethacin, ibuprofen, flurbiprofen, naproxen, and wogonin) as axial ligands have been reported in literature [27,28,29,30,31,32,33,34,35].

In the present work, two complexes containing axial ketoprofen (2-(3-benzoylphenyl)propanoic acid) or naproxen (2-(6-methoxynaphthalen-2-yl)propanoic acid) (Figure 2) were synthesized and tested for their biological features against a panel of human cancer cell lines. Ketoprofen and naproxen are two classic NSAID propionic acid derivatives, acting as inhibitors of both COX-1 and COX-2 [23,36]. The presence of a carboxylic functionality in their structure allows an easy coordination to the Pt(IV) core through an “esterification” reaction of the hydroxido synthon [37].

## 2. Results and Discussion

### 2.1. Synthesis and Characterization of the Pt(IV) Complexes

**Figure 3:**
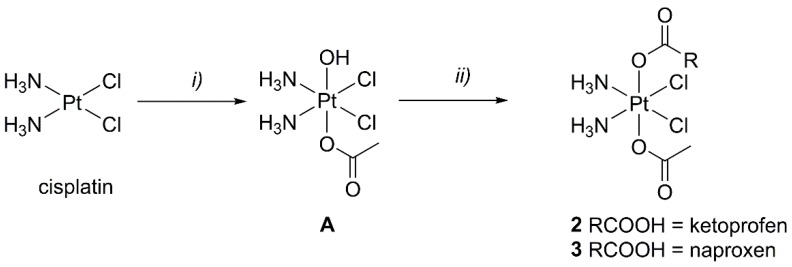
Scheme of the syntheses used in this work: (**i**) 50% *w*/*w* H_2_O_2_ in CH_3_COOH (room temperature, 4 h), (**ii**) acyl chloride of non-steroidal anti-inflammatory drugs (NSAIDs) in acetonitrile, under microwave heating (55 °C, 1 h).

The synthesis of complexes **2** and **3** began with the preparation of the intermediate (*OC*-6-44)-acetatodiamminedichloridohydroxidoplatinum(IV) (**A**, Figure 3) from cisplatin and hydrogen peroxide in acetic acid according to published procedures [38]. The following step required the conversion of the carboxyl group of NSAIDs into its more reactive acyl chloride using oxalyl chloride and *N*,*N*-dimethylformamide (DMF) as a catalyst. The acyl chlorides of the NSAIDs were then coordinated to the Pt core by a microwave-assisted reaction with **A** in anhydrous acetonitrile with a small amount of pyridine (able to neutralize the hydrochloric acid released during the reaction) to obtain **2** and **3**. The microwave heating was applied because there is neat advantage in terms of reaction speed, yield, and the purity of products, with respect to the traditional thermal syntheses. This improvement is well-known in organic chemistry, but it has been seldom reported in the syntheses of Pt(II) and Pt(IV) complexes [39,40,41,42]. The synthesis of complex **3** has been already reported in the literature, but this was done with a different procedure [35].

Finally, as a reference compound, **1** was prepared, according to the procedure reported by Dhar et al., by reacting (*OC*-6-44)-diamminedichloridodihydroxidoplatinum(IV) with the anhydride of acetylsalicylic acid [24].

All the complexes were characterized with reversed-phase high performance liquid chromatography (RP-HPLC) coupled with electrospray ionization-mass spectrometry (ESI-MS) and multinuclear nuclear magnetic resonance (NMR; see Figures S1–S13, Supplementary Material). Their lipophilicity was evaluated by RP-HPLC measurements [43]. Indeed, the chromatographic retention was due to partitioning between C18 chains of the stationary phase (a model of the cell membrane) and the aqueous eluent (a model of the water inside and outside cells). The logarithm of the RP-HPLC capacity factor *k*′ (*k*′ = (*t*_R_ − *t*_0_)/*t*_0_—where *t*_R_ = retention time of the compound under investigation and *t*_0_ = column dead time)—usually correlates with the logarithm of the octanol/water partition coefficient, log *P*_o/w_ (typically employed to represent lipophilicity). The log *k*′ values for the complexes under investigation were measured on a C18 HPLC column by using a 70% methanol/30% aqueous 15 mM formic acid eluent. In these conditions (high methanol content), the *t*_R_, and consequently the log *k*’, are compressed, but maintain the correct trend. Therefore, the aspirin-containing **1** results tended to be the most hydrophilic of the series, while naproxen-containing **3** was slightly more lipophilic than ketoprofen-containing **2**, as expected from the log *P*_o/w_ values of the free NSAIDs (log *P*_o/w_ = 1.13 for aspirin, 2.77 for ketoprofen, and 3.06 for naproxen) [44].

Despite the “traditional” claim that Pt(IV) complexes are inert, several members of this family have shown changes in the coordination sphere when challenged in a solution [6]. Therefore, compounds **2** and **3** were dissolved in a 2 mM HEPES (HEPES = 4-(2-hydroxyethyl)-1-piperazineethanesulfonic acid, pH = 7.5) buffer with 5% *v*/*v* ethanol to improve the solubility, and the HPLC traces were recorded up to 7 days. All compounds showed a very low decomposition during the experiment (from 24 h to 7 days: **2** 94.5% to 79.8%; **3** 98.3% to 75.9% vs. the original peak). In both cases, the ESI-MS spectra showed that the main by-product derived from the chloride/water exchange in the equatorial position of the original octahedral assembly.

The redox properties of the Pt(IV) complexes could be evaluated through their reduction potential. However, a suitable (high) potential may not guarantee their in vivo reduction, as the kinetics plays an important role [9]. Since the high molecular weight cytosolic bioreductants mainly reduce the Pt(IV) complexes [9,45], such reduction was studied via NMR using ^15^NH_3_-labeled complexes. Usually, the [^1^H, ^15^N] the HSQC spectra of ^15^N-cisplatin-based Pt(IV) complexes when reduced in cytosol show only the presence of cisplatin (^15^NH_3_ *δ* = –66.7 ppm and ^1^H *δ* = 4.0 ppm, with satellite peaks possibly visible at −63.7 ppm and −69.6 ppm, ^1^*J*_Pt-N_ = 331 Hz and ^2^*J*_Pt-H_ = 63 Hz) [41,46,47]. The production of cisplatin was verified for both ^15^N-**2** and^15^N-**3** (synthesized according to the actual procedures from ^15^N-cisplatin) [38,48] after 2 h of treatment with cytosol extracted from A2780 ovarian cancer cells (data not shown).

### 2.2. Antiproliferative Activity

The half-maximal inhibitory concentrations, IC_50_, were determined for **2** and **3**, along with the clinically-employed reference complexes cisplatin and oxaliplatin, as well as asplatin **1**, free ketoprofen, and naproxen for comparison purposes (Table 1). Six human cancer cell lines were challenged with the compounds under investigation: Lung carcinoma A-549, colon adenocarcinoma HT-29, HCT 116, SW480, ovarian endometroid adenocarcinoma A2780, and malignant pleural mesothelioma (MPM) MSTO-211H (a mixed or biphasic phenotype consisting of both epithelioid and sarcomatoid components). The MPM cell line was added to the panel, since promising preclinical results have been reported on the activity of celecoxib on MPM, albeit apparently independent of COX-2 protein levels [49].

**Figure 4:**
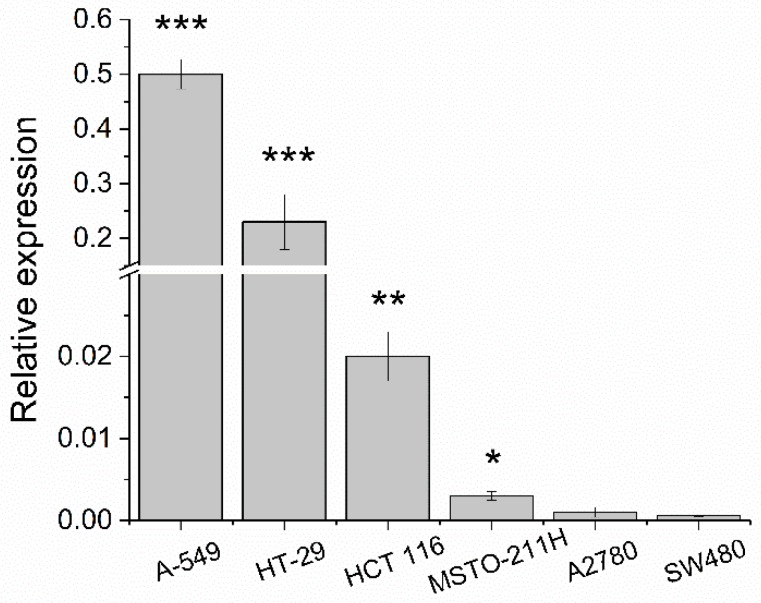
Cyclooxygenase (COX)-2 cDNA level of expression (ΔCq) in A-549, HT-29, HCT 116, MSTO-211H, A2780, and SW480 cells. Data are means ± SEM (standard error of mean) of at least three independent replicates and were compared by means of a one-way ANOVA analysis of the variance-Tukey test (no indication = not significant; * *p* < 0.05; ** *p* < 0.01; *** *p* < 0.001 vs. SW480).

In these cell lines, the level of COX-2 expression was evaluated just before the viability tests by a quantitative reverse transcription polymerase chain reaction (RT-qPCR) [50,51], resulting observably high in A-549 and HT-29, medium in HCT 116, low in MSTO-211H, and very low in A2780 and SW480 (Figure 4). A significant amount of amplicon was found in all samples (i.e., the cycle quantification value C_q_ within the dynamic linear range of the standard curve. See Section 4).

At the first glance, the Pt(IV)-NSAID complexes **2** and **3** appear to have been more active than cisplatin, oxaliplatin, and **1** on all cell lines tested ranging from 2 to 24 times. Moreover, naproxen-based **2** was (almost always) slightly more active than ketoprofen-based **3**.

To extract quantitative trends from the bulk of the IC_50_ data, the ratio R of the activity of cisplatin-based Pt(IV) derivatives **1**, **2**, and **3** vs. the activity of parent cisplatin against each cancer cell was calculated and inserted in Table 1. Since the antiproliferative activity is the inverse of the corresponding IC_50_ value, it follows that: R = IC_50_ (cisplatin)/IC_50_ (Pt(IV) conjugate). What is noteworthy is that the trend of the R values does not parallel the expression of COX-2. In particular, within the colon cancer family, the HCT 116 and SW480 cells, which have lower level of COX-2 with respect to HT-29, resulted more sensitive to **2**–**3** treatments. From this scenario, the antiproliferative activity seems to be more related to the lipophilicity of the complexes (log *k’*) than to the COX-2 expression in these tumor cell lines. In addition, the IC_50_ values of the free NSAIDs ketoprofen and naproxen resulted to be quite similar (in the low mM range) and seemed to be unrelated to the COX-2 expression.

**Figure 5:**
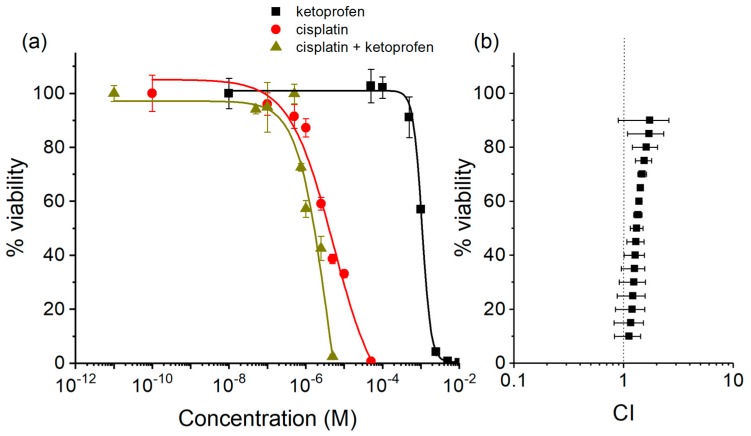
(**a**) Residual HCT 116 viability data and concentration response curves (four parameters logistic function) after 72 h of treatment of cisplatin (red circles), ketoprofen (black squares), and a mixture of cisplatin and ketoprofen (final cisplatin:ketoprofen ratio = 1:500, dark yellow triangles). Data are means ± SEM of at least three replicates. (**b**) Residual viability data for the 1:500 mix were used to obtain the Combination Index (CI) value by using the equation of Chou and Talalay for non-mutually exclusive drugs (see Materials and Methods).

Moreover, the combination index (CI) values [52,53,54] between free cisplatin and the two NSAIDs were calculated for HT-29 and HCT 116, showing a high and medium COX-2 expression, respectively. In all cases, the CI pointed out an almost additive combination (CI around 1), once again independently from COX-2 expression (Figure 5 and Figures S14–S17, Supplementary Material).

**Figure 6:**
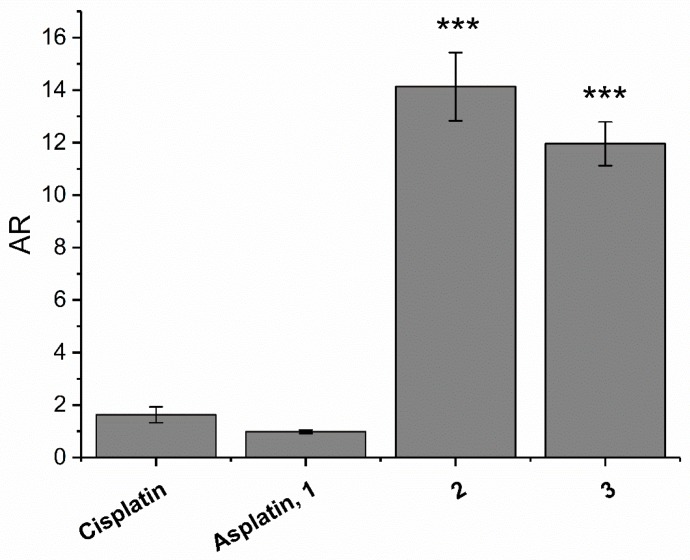
Accumulation ratio (AR) of cisplatin, **1**, **2**, and **3**, measured on A2780 ovarian cancer cells treated for 4 h with 10 μM concentrations of the Pt complexes. Data are means ± SEM of at least three independent replicates. Data are means ± SEM of at least three independent replicates and were compared by means of a one-way ANOVA analysis of a variance-Tukey test (no indication = not significant; *** *p* < 0.001 vs. cisplatin).

The intracellular Pt accumulation of **1**–**3** and cisplatin was assessed to verify the relationship existing with their lipophilicity (log *k*’). This parameter, which is the final balance between cellular influx and efflux [55], is here expressed as the accumulation ratio (AR), the ratio between the intracellular [Pt] and the extracellular [Pt] (in the culture medium) [56]. For this purpose, A2780 cells were treated for 4 h with 10 μM concentrations of all Pt complexes, and the AR values were calculated from [Pt] experimentally measured by inductively coupled plasma-mass spectrometry (ICP-MS) (Figure 6). The AR trend paralleled the differences in antiproliferative activity (1/IC_50_) between the group formed by **1** and cisplatin and that formed by **2** and **3**.

In dicarboxylato Pt(IV) complexes, the combination of (hydrophilic) cisplatin and two (amphiphilic) carboxylate anions causes the neutralization of their negative charges while the lipophilic chains protrude toward the exterior, thus generating very lipophilic conjugates. These combo Pt(IV) prodrugs enter cells via passive diffusion (the main if not the only mechanism of their cellular uptake) more efficiently than the single components (synergistic cellular accumulation) [57]. 

### 2.3. Gene Modulation and RT-qPCR

The apparent absence of relationship between *COX-2* expression and sensitivity towards free ketoprofen and naproxen or **2** and **3** suggests that these drugs might inhibit cell growth through a COX-independent mechanism.

**Figure 7:**
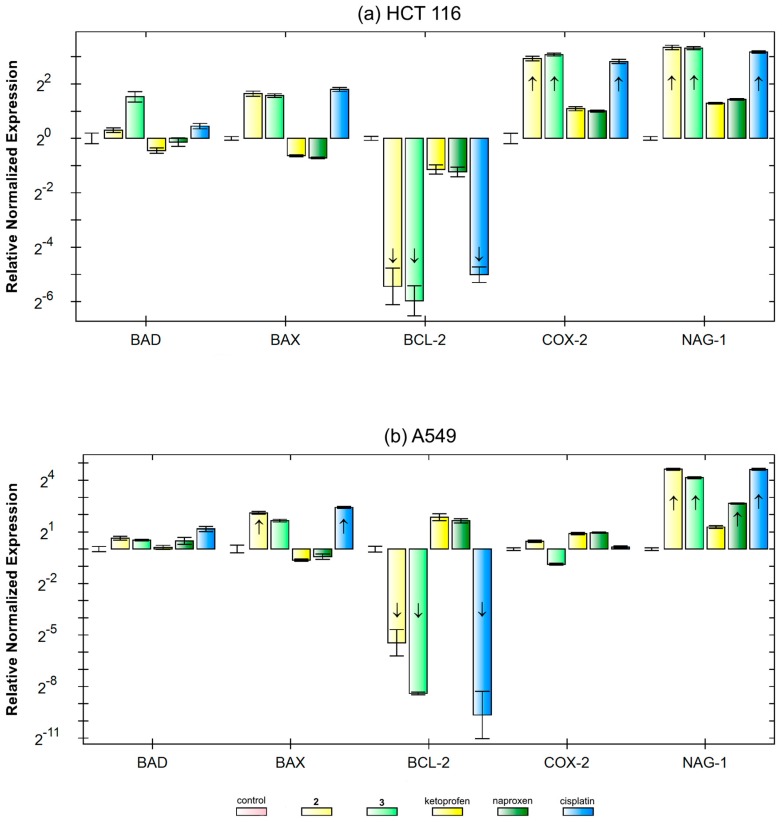
Relative gene expression of *BAD* (pro-apoptotic), *BAX* (pro-apoptotic), *BCL-2* (anti-apoptotic), *COX-2* (cyclooxygenase-2), and *NAG-1* (NSAID-activated gene 1) in (**a**) HCT 116 (lower *COX-2* expression) and (**b**) A-549 (higher *COX-2* expression) cells following equitoxic 24 h treatments (IC_50_) with cisplatin, **2**, **3**, ketoprofen, and naproxen. From the left to right of each sequence: Control, **2** (light yellow), **3** (light green), ketoprofen (dark yellow), naproxen (dark green), and cisplatin (light blue). The RT-qPCR experiment was performed in triplicate, and the results were normalized to references genes in untreated control. The arrows indicate the upregulated (arrow up) or downregulated (arrow down) gene expression with respect to internal threshold value as determined by the CFX Manager software.

To shed more light on the underlying biochemical mechanism, a RT-qPCR analysis was carried out. Preliminarily, to perform equitoxic treatments, the IC_50_ values after 24 h of continuous treatment (the time interval of the corresponding RT-qPCR experiment) were obtained. In this experiment, the variation of the expression of five genes (i.e., *BAD*, *BAX*, *BCL-2*, *COX-2*, and *NAG-1*) was analyzed. Two cell lines, namely A-549 (high *COX-2* expression) and HCT 116 (low *COX-2* expression), were chosen for this experiment (Figure 7).

Apoptosis, the phenomenon of programmed cell death, is triggered through two major routes: The extrinsic and the intrinsic (mitochondrial) pathways. The second mechanism is regulated by the B cell leukemia-2 gene product (*BCL-2*) family. This family contains both pro-apoptotic (e.g., *BAX*, *BAK*, and *BAD*) and anti-apoptotic (e.g., *BCL-2* and *BCL-X_L_*) members [58]. Agents able to increase the levels of pro-apoptotic proteins or decrease that of anti-apoptotic proteins are good anticancer candidates.

As already reported in the introduction, albeit with the acknowledgment that the anti-tumorigenic mechanism of NSAIDs is not fully understood, both COX-dependent and -independent pathways seem to play a role. Recent studies have revealed the existence of the NSAID activated gene (*NAG-1*), a member of the transforming growth factor beta, *TGF-β*, superfamily [59,60], involved in antiproliferative activity [61,62]. Importantly, *NAG-1* is induced by NSAIDs in cells also devoid of any COX activity [63,64] The induced expression of *NAG-1* can thus be correlated with the growth inhibition of cancer cells that follows a COX-independent mechanism [18,65].

Cisplatin **2** and **3** showed similar effects on both anti-apoptotic and pro-apoptotic genes in both cell lines, indicating that cisplatin generated by Pt(IV)→Pt(II) reduction plays the paramount role. Their major effect was the downregulation of anti-apoptotic *BCL-2*, more intensely on A-549 than HCT 116, and the up-regulations of pro-apoptotic *BAD* and *BAX*. On the contrary, these contrasting but negligible effects (i.e., no changes compared to regulation threshold) were caused by free ketoprofen and naproxen. Pt(IV) conjugates and free NDAIDs did not significantly change the COX-2 expression on A-549 cells, whereas it smoothly increased it on HCT 116 cells with a substantial contribution of cisplatin to increase the expression of *COX-2*. 

In order to confirm the RT-qPCR data (i.e., the *COX-2* expression remained almost unchanged on A-549 cell lines or even slightly increased on HCT 116), a semi-quantitative immunochemical analysis was carried out using a primary antibody against COX-2. Indeed, the fluorescence microscopy indicated a similar amount of COX-2 protein in the nuclear region after 24 h treatment with equitoxic concentrations of **2** and **3**. Only a slight decrease in cytosolic staining was observed in treated A-549 cells, and this was attributed to the inactive form of the enzyme (see Figures S18 and S19, Supplementary Material) [66,67].

Interestingly, **2** and **3** and, to a lesser extent, free ketoprofen and naproxen increased the expression of the above-discussed *NAG-1*. In addition, cisplatin increased *NAG-1* expression, thus augmenting the overall NAG-1 stimulation.

## 3. Materials and Methods

### 3.1. General Procedures

All the chemicals (Sigma Aldrich-Merck KGaA, Darmstadt, Germany or Alfa Aesar–Thermo Fisher GmbH, Kandel, Germany) were used without further purification. The complex (OC-6-44)-acetatodiamminedichloridohydroxidoplatinum(IV) (**A**) was prepared according to previously published procedures [38,68]. The reactions under microwave heating were performed by using a CEM Discover^®^ SP System equipped with a focused single mode, a self-tuning cavity, an air-cooling system, and an automated power control based on temperature feedback that supplied power in 1 W increments from 0 to 300 W.

The purity of all the synthesized compounds was assessed by analytical RP-HPLC. Chromatographic analyses were carried out using a C18 Phenomenex Phenosphere-NEXT (5-μm, 250 × 4.6 mm internal diameter) column on a Waters HPLC-MS instrument (equipped with Alliance 2695 separations module, 2487 dual lambda absorbance detector, and 3100 mass detector). The UV-visible detector was set at 210 nm, the eluent was a 30/70 mixture of 15 mM formic acid/CH_3_OH, and the flow rate was 0.5 mL·min^−1^. Mass spectra were recorded using source and desolvation temperatures set to 150 °C and 250 °C, respectively, with nitrogen used as both drying and nebulizing gas. The cone and the capillary voltages were usually +30 V (positive ion mode) or −30 V (negative ion mode) and 2.70 kV, respectively. The quasi-molecular ion peak [M + H]^+^ of the synthesized complexes was assigned on the basis of the *m/z* values and of the simulated isotope distribution patterns. The NMR spectra were measured on a NMR-Bruker Avance III spectrometer operating at 500 MHz (^1^H), 125.7 MHz (^13^C), and 107.2 MHz (^195^Pt). ^1^H and ^13^C NMR chemical shifts were reported in parts per million (ppm) and referenced to solvent resonances. ^195^Pt NMR spectra were recorded using a solution of K_2_[PtCl_4_] in saturated aqueous KCl as the external reference. The shift for K_2_[PtCl_4_] was adjusted to −1628 ppm from Na_2_PtCl_6_ (*δ* = 0 ppm).

### 3.2. Synthesis of (RS)-2-[3-(benzoyl)phenyl]propanoyl Chloride (Acyl Chloride of Ketoprofen)

(*RS*)-2-[3-(benzoyl)phenyl]propanoic acid (0.034 g, 0.134 mmol) was placed into a 10 mL flask and dissolved in 2 mL of dichloromethane; then, 0.115 mL of oxalyl chloride (1.34 mmol) were added together with a drop of dimethylformamide. After the end of gas development, the transparent and colorless mixture was allowed to react at room temperature overnight. The yellow solution was dried by a rotary evaporator at 60 °C obtaining a yellow–green oil. ^1^H-NMR (CDCl_3_): 1.52 (3H, d, *J* = 7.2 Hz, H3), 3.80 (1H, q, *J* = 7.2 Hz, H2), 7.40–7.47 (3H, m, H5, H6, H9), 7.56 (2H, t, *J* = 7.7 Hz, H13, H15), 7.64 (1H, d, *J* = 7.7 Hz, H7), 7.78 (3H, m, H12, H14, H16) ppm; ^13^C-NMR (CDCl_3_): 18.7 (C3), 57.2 (C2), 128.5 (C13, C15), 129.1 (C6), 129.6 (C7), 129.9 (C12, C16), 130.1 (C9), 131.8 (C14), 132.8 (C5), 137.3 (C4), 137.9 (C11), 138.4 (C8), 175.3 (C1), 196.1 (C10) ppm.

### 3.3. Synthesis of 2-(6-metoxynaphtalen-2-yl)propanoyl Chloride (Acyl Chloride of Naproxen)

2-(6-metoxynaphtalen-2-yl)propanoic acid (0.030 g, 0.132 mmol) was placed into a 10 mL flask and dissolved in 2 mL of dichloromethane; then, 0.114 mL of oxalyl chloride (1.32 mmol) were added together with a drop of dimethylformamide. After the end of gas development, the transparent and colorless mixture was allowed to react at room temperature overnight. The yellow solution was dried by a rotary evaporator at 60 °C obtaining a yellow–green oil. ^1^H-NMR (CDCl_3_): 1.62 (3H, d, *J* = 7.0 Hz, H3), 3.87 (3H, s, H14), 4.22 (1H, q, *J* = 7.0Hz, H2), 7.10 (1H, s, H9), 7.13 (1H, m, H5), 7.30 (1H, d, *J* = 8.4 Hz, H11), 7.64 (1H, s, H13), 7.69 (2H, m, H6, H10) ppm; ^13^C-NMR (CDCl_3_): 18.7 (C3), 55.4 (C14), 57.4 (C2), 105.7 (C13), 119.4 (C11), 125.9 (C6), 127.0 (C9), 127.7 (C5), 128.9 (C8), 129.4 (C10), 132.5 (C7), 134.2 (C4), 158.1 (C12), 175.7 (C1) ppm.

### 3.4. Synthesis of (OC-6-44)-acetatodiamminedichlorido(RS)-2-[3-(benzoyl)phenyl]propanoatoplatinum(IV) (**2**)

(*OC*-6-44)-acetatodiamminedichloridohydroxoplatinum(IV) **A** (0.050 g, 0.134 mmol) was suspended in 2 mL of anhydrous acetonitrile in a microwave tube. (*RS*)-2-[3-(benzoyl)phenyl]propanoyl chloride was then dissolved in 1 mL of acetonitrile which was added to the suspension together with 30 μL of pyridine. The mixture was allowed to react in a microwave reactor for one hour, at 55 °C, with a power of 80 W. The resulting yellow–green solution was filtered (polytetrafluoroethylene filter, porosity: 0.45 μm) to remove unreacted reagents, and then it was dried with a rotary evaporator. The resulting residue was dissolved in 2 mL of dichloromethane, and then 10 mL of diethyl ether were added to get the precipitation of a yellow solid. The precipitate was separated by centrifugation and washed several times with diethyl ether, water and, finally, diethyl ether again, drying the residue under nitrogen flow before any change of solvent and at the end. Yield: 0.043 g, 52%. ESI-MS: found 613 *m/z;* calcd for C_18_H_23_Cl_2_N_2_O_5_Pt, [M + H]^+^ 613 *m/z*; ^1^H-NMR (DMSO-d_6_): 1.36 (3H, d, *J* = 7.2 Hz, H5), 1.91 (3H, s, H2), 3.80 (1H, q, *J* = 7.2 Hz, H4), 6.50 (6H, m, NH_3_), 7.47 (1H, t, *J* = 7.6 Hz, H8), 7.56–7.70 (6H, m, H7, H9, H11, H15, H16, H17), 7.75 (2H, d, *J* = 7.6 Hz, H14, H18) ppm; ^13^C-NMR (DMSO-d_6_): 19.8 (C5), 22.8 (C2), 46.2 (C4), 127.8 (C8), 128.3 (C9), 128.6 (C15, C17), 128.8 (C11), 129.7 (C14, C18), 132.1 (C16), 132.6 (C7), 136.8 (C13), 137.0 (C10), 142.6 (C6), 178.1 (C1), 181.4 (C3), 195.7 (C12) ppm; ^195^Pt-NMR (DMSO-d_6_): 1214 ppm.

### 3.5. Synthesis of (OC-6-44)-acetatodiamminedichlorido2-(6-metoxynaphtalen-2-yl)propanoatoplatinum(IV) (**3**)

(*OC*-6-44)-acetatodiamminedichloridohydroxidoplatinum(IV) (0.050 g, 0.133 mmol) was suspended in 2 mL of anhydrous acetonitrile in a microwave tube. 2-(6-metoxynaphtalen-2-yl)propanoyl chloride was dissolved in 1 mL of acetonitrile and added to the suspension together with 30 μL of pyridine. The mixture was allowed to react in a microwave reactor for one hour, at 55 °C, with a power of 80 W. The resulting yellow solution was filtered with a polytetrafluoroethylene filter (porosity: 0.45 μm) to remove unreacted reagents, and it was subsequently dried by a rotary evaporator obtaining a light-yellow residue. The complex was precipitated by adding 2 mL of dichloromethane and then 10 mL of diethyl ether, causing the formation of a sticky yellow solid. The solid residue was tritured with diethyl ether to get a yellow powder and then dried under nitrogen flow. The residue was further washed with water, dried under nitrogen flow, and kept in desiccator. Yield: 0.052 g, 65%. ESI-MS: found 589 *m/z*; calcd for C_16_H_23_Cl_2_N_2_O_5_Pt, [M + H]^+^ 589 *m/z*. ^1^H-NMR (DMSO-d_6_): 1.40 (3H, d, *J* = 7.2 Hz, H5), 1.91 (3H, s, H2), 3.81 (1H, q, *J* = 7.2 Hz, H4), 3.86 (3H, s, H16), 6.53 (6H, m, NH_3_), 7.13 (1H, m, H7), 7.27 (1H, m, H13), 7.47 (1H, m, H15), 7.73 (3H, m, H8, H11, H12) ppm; ^13^C-NMR (DMSO-d_6_): 19.8 (C5), 22.8 (C2), 46.4 (C4), 55.1 (C16), 105.7 (C15), 118.3 (C13), 125.4 (C8), 126.3 (C11), 126.9 (C12), 128.3 (C10), 129.1 (C7), 133.0 (C9), 137.4 (C6), 156.9 (C14), 178.2 (C1), 181.9 (C3) ppm; ^195^Pt-NMR (DMSO-d_6_): 1217 ppm.

### 3.6. Lipophilicity and Stability in Water

A chromatogram for each complex (0.25 mM) at a fixed eluant composition (70% methanol/30% aqueous 15 mM formic acid) was performed on a C18 column (Phenomenex Phenosphere-NEXT, 5 μm, 250 × 4.6 mm internal diameter). The corresponding retention time *t*_R_ was used to calculate log *k*′:*k*′ = (*t*_R_ − *t*_0_)/*t*_0_, where *t*_0_ is the column dead time (i.e., the *t*_R_ of KCl used as an internal reference) [46,69,70].

The stability in water of the compounds was checked by dissolving the Pt complexes ([Pt] = 0.5 mM) in a 2 mM HEPES buffer (HEPES = 4-(2-hydroxyethyl)-1-piperazineethanesulfonic acid, pH = 7.5) containing 5% *v*/*v* ethanol as a cosolvent. The HPLC traces (see above for the instrumental conditions) were recorded along one week.

### 3.7. Antiproliferative Activity

The compounds under investigation were tested on ovarian endometrioid adenocarcinoma A2780 (ICLC HTL98008 from Interlab Cell Line Collection, ICLC, Genova, Italy), lung adenocarcinoma A-549 (ICLC HTL03001), biphasic malignant pleural mesothelioma MSTO-211H (ICLC HL01018), colon carcinoma HCT 116 (ECACC 91091005 from European Collection of Cell Cultures, ECACC, Salisbury, UK), and both HT-29 and SW480 (a kind gift from Prof. Claudia Cantoni, University of Genova, Italy). An RPMI 1640 medium as used for the A2780, A-549, and SW480 cells, McCoy’s 5A medium was used for HCT 116 and HT-29 cells, and Dulbecco’s Modified Eagle Medium (DMEM) was used for MSTO-211H. All media contained L-glutamine (2 mM) and were supplemented with penicillin (100 IU·mL^−1^), streptomycin (100 mg·L^−1^) and 10% heat inactivated fetal bovine serum (FBS). Cell culture and treatment were carried out at 37 °C in a 5% CO_2_ humidified chamber. Cisplatin was dissolved in a 0.9% *w*/*v* NaCl aqueous solution brought to pH 3 with HCl (final stock concentration 1 mM). All Pt(IV) complexes were dissolved in absolute ethanol (final stock concentration 1–5 mM) and stored at −20 °C. The mother solutions were diluted in a complete medium to the required concentration range, and, where present, the total co-solvent concentration never exceeded 0.2% (this concentration was found to be non-toxic to the cells tested). The concentration was confirmed by means of inductively coupled plasma-optical emission spectroscopy (ICP-OES). Ketoprofen and naproxen were freshly dissolved in 0.1 M NaHCO_3_ (final stock concentration 5 and 2.5 mM, respectively). Cells were treated with the compounds under investigation for 72 h. To assess the growth inhibition of the compounds under investigation, a cell viability test (i.e., the resazurin reduction assay) was used. Briefly, 2 × 10^3^ cells per well were seeded in black sterile tissue-culture treated 96-well plates. At the end of the treatment, viability was assayed by 100 μg·mL^−1^ resazurin (Acros Chemicals-Thermo Fisher Scientific, Geel, Belgium) in a fresh medium for 1 h at 37 °C, and the amount of the reduced product, resorufin, was measured by means of fluorescence (excitation *λ* = 535 nm, emission *λ* = 595 nm) with a Tecan Infinite F200Pro plate reader (Tecan, Grödig, Austria) [71]. In each experiment, cells were challenged with the drug candidates at different concentrations, and the final data were calculated from at least three replicates of the same experiment performed in triplicate. The fluorescence of 8 wells containing medium without cells were used as a blank. Fluorescence data were normalized to 100% cell viability for non-treated cells. The half-maximal inhibitory concentration (IC_50_), defined as the concentration of the drug reducing cell viability by 50%, was obtained from the dose-response sigmoid using Origin Pro (version 8, Microcal Software, Inc., Northampton, MA, USA). In order to verify the synergy between NSAIDs and cisplatin, HCT 116 and HT-29 cells were treated with increasing concentrations of cisplatin, ketoprofen or naproxen, and a mixture cisplatin:NSAIDs in a fixed 1:500 ratio, according to their respective IC_50_ values. The experiment was repeated three times. According to the method of Chou and Talalay [52,53,54], the interaction between cisplatin and NSAIDs was computed in terms of combination index (CI) for non-mutually exclusive drugs by using the following equation:

(1) $$CI=\frac{C1_{m}}{C1_{a}}+\frac{C2_{m}}{C2_{a}}+\frac{C1_{m}C2_{m}}{C1_{a}C2_{a}}$$

where *C*1 and *C*2 are the drug concentrations used in the mix (*C*1*_m_* and *C*2*_m_*) or alone (*C*1_a_ and *C*2_a_) to obtain the same level of residual viability. Based on the actual experimental data, the CI values were calculated by solving the equation over an entire range of residual viability (from 5% to 95%, obtained from the sigmoidal regression). These data were then used to generate residual viability vs. CI plots, which is an effect-oriented means of presenting synergism or antagonism. The interpretation of CI values is defined such that CI = 1 indicates an additive effect, and CI < 1 and CI > 1 indicate synergism and antagonism, respectively.

### 3.8. Cellular Uptake

A2780 cells were seeded in T25 flasks and allowed to grow until around 80% confluence. Then, the treatment was performed for 4 h with the complexes under investigations (10 μM) in a complete medium. At time zero, 100 μL of medium was taken out from each sample to check the extracellular Pt concentration. At the end of the exposure, cells were washed three times with phosphate buffered saline (PBS), detached from the Petri dishes using 0.05% Trypsin 1× + 2% EDTA (HyClone^TM^-, Thermo Fisher Scientific, Waltham, MA, USA) and harvested in a fresh complete medium. The cell pellet was lysed in 1% *w*/*v* sodium dodecyl sulphate and the protein content was determined by means of the bicinchoninic acid method (BCA assay Kit, Pierce^TM^, Thermo Fisher, Waltham, MA, USA). The exact volume of the sample was determined by weight. The cell number was computed by a standard curve with known amount of the same cells. Control and cisplatin-treated cells showed the same values as those previously reported. Before the ICP-MS measurement, the HNO_3_ was diluted to a final 1% concentration. To obtain the Pt cellular concentration, the total cellular volume of each sample was obtained considering the mean cell diameter and cell number estimated by means of an automatic cell counting device (Countess^®^, Life Technologies-Thermo Fisher, Waltham, MA, USA). The ratio between the internal and the external cell Pt concentration, namely, the accumulation ratio (AR) was computed as previously reported [72].

### 3.9. Quantitative Reverse Transcription PCR (RT-qPCR)

HCT 116 and A-549 cells (2 × 10^6^) were seeded on T25 flasks and allowed to attach for 24 h. The treatment was performed with equitoxic concentrations (i.e., HCT 116: 3 μM for both complexes **2** and **3**, 1 mM for both ketoprofen and naproxen, and 30 μM for cisplatin; A-549: 5 μM for both complexes **2** and **3**, 1 mM for both ketoprofen and naproxen, and 50 μM for cisplatin). After 24 h, RNA was extracted and purified by DNAse treatment with a commercial kit (RNASPIN MINI, GE Healthcare, Pittsburgh, PA, USA); then it was quantified and checked for purity by means of absorbance at λ = 260, 280, and 340 nm in UV-Star^®^ half-area UV transparent plate (Fisherbrand-Thermo Fisher, Waltham, MA, USA) with the above-mentioned microplate reader. For each sample, 1 μg of RNA was retrotranscribed (RT) to cDNA with the Revertaid cDNA First strand kit (Thermo Fisher, Waltham, MA, USA) using random hexamer primers at 45 °C, following the manufacturer’s instructions. qPCR was performed in triplicate on each sample (10 ng) to detect the expression levels of *BAD*, *BAX*, *COX-2*, *NAG-1*, and the reference genes RNA18S, HPRT1, and GAPDH. Primer sequences were designed using the NCBI tool and checked for target specificity, including splice variants (see Supplementary Material, Table S1). Reactions were based on the (PowerUp SYBR^TM^, Thermo-Fisher) in the presence of 0.4 μM primer pairs except for RNA18S (0.2 μM), according to the manufacturer’s instructions, in a reaction volume of 10 μL. In order to compute reaction efficiency, a standard curve was performed for each master mix. qPCR was performed in triplicate using and the CFX368 thermal cycler (Bio-Rad, Hercules, CA, USA). The reaction conditions were 95 °C for 1 min, followed by 45 cycles at 98 °C for 5 s, and the annealing–extension step for 5 s at 60 °C, with data collection. At the end of these cycles, a melting curve (65–95 °C, with the plate read every 0.5 °C) was performed in order to assess the specificity of the amplification product by single peak melting temperature verification. Results were normalized on the reference genes and on the control according to the ΔCq and ΔΔCq method, respectively [50]. All data analyses were performed with the built-in software (CFX Manager, Bio-Rad, Hercules, CA, USA).

## 4. Conclusions

**Figure 8:**
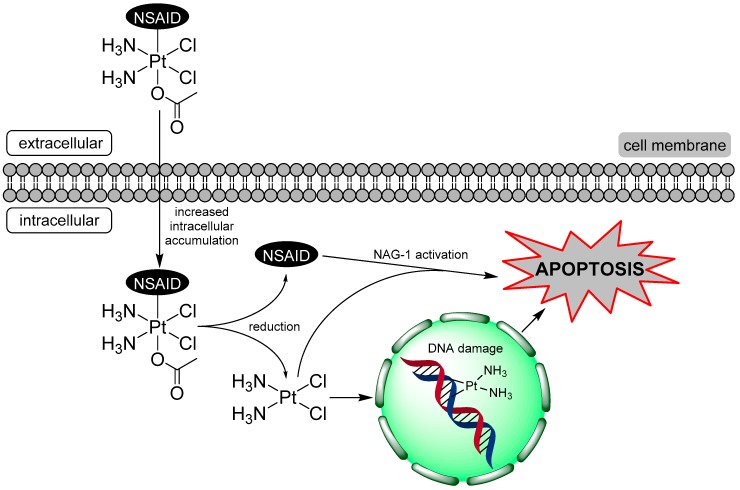
Proposed mechanism of action of the Pt(IV)-NSAID complexes.

The Pt(IV)-NSAID complexes **2** and **3** showed higher antiproliferative activity on the cancer cell lines under investigation when compared to cisplatin, **1,** and the corresponding free NSAIDs, ketoprofen and naproxen. The trend of activity can be explained in terms of lipophilicity of the compounds (evaluated here by means of their log *k’*), that affects their cellular accumulation. This is often the main reason of the increased activity of most Pt(IV) conjugates, defined as synergistic cellular accumulation. On the contrary, the trend of activity seems to be scarcely dependent on *COX-2* gene expression, suggesting that a COX-2-independent mechanism may play a role (Figure 8). This mechanism has been tentatively identified in the activation of NAG-1, a protein that has antitumorigenic and pro-apoptotic propensity, linked, at least in part, to the chemoprevention activity of NSAIDs [59]. However, analyzing the variation of pro-apoptotic *BAD* and *BAX* and anti-apoptotic *BCL-2* genes, it appears clear that cisplatin, released after Pt(IV) → Pt(II) reduction, is the component of the combos mainly responsible for the overall antiproliferative activity—also because of its own *NAG-1* activation. In conclusion, most of the biological effects are related to the cisplatin metabolite. The NSAID axial ligands increase the overall lipophilicity and, hence, the cellular accumulation of these combos, offering a minor (additive) contribution in terms of *NAG-1* activation.

## Figures and Tables

**Table 1:** Lipophilicity (log *k*’) and antiproliferative (IC_50_) data for complexes **1**–**3**, together with cisplatin, oxaliplatin, aspirin, ketoprofen, and naproxen. IC_50_ data (μM) were obtained after 72 h of treatment of the following cancer cell lines: Lung A-549, colon HT-29, HCT 116, SW480, ovarian A2780 cancer cell lines, and malignant pleural mesothelioma MSTO-211H. Data are means ± standard deviation (SD) of at least three independent replicates. Numbers in parenthesis are the ratios R = IC_50_ (cisplatin)/IC_50_ (Pt(IV) conjugate) reported with two significant figures.

Compounds	log *k*’	IC_50_ (μM)					
		A-549	HT-29	HCT 116	MSTO-211H	SW480	A2780
cisplatin	−0.50	3.60 ± 0.90	2.72 ± 0.39	3.05 ±0.28	1.33 ± 0.35	2.27 ± 0.12	0.460 ± 0.110
oxaliplatin	−0.28	0.74 ± 0.25	0.92 ± 0.08	1.16 ± 0.09	1.01 ± 0.55	0.48 ± 0.02	0.171 ± 0.008
aspirin	−0.08	1672 ± 152	2835 ± 885	2578 ± 772	639 ± 374	1607 ± 386	1597 ± 455
ketoprofen	0.40	725 ± 69	828 ± 229	984 ± 179	518 ± 296	830 ± 173	676 ± 36
naproxen	0.50	701±28	764 ± 158	640 ± 91	427 ± 159	927 ± 257	353 ± 64
asplatin, **1**	−0.32	6.4 ± 2.7 (0.56)	4.42 ± 0.21 (0.62)	1.50 ± 0.083 (2.0)	1.74 ± 0.21 (0.75)	0.217 ± 0.07 (1.1)	0.552 ± 0.123 (0.83)
**2**	0.14	0.825 ± 0.388 (4.4)	0.486 ± 0.235 (5.6)	0.184 ± 0.088 (17)	0.198 ± 0.035 (6.7)	0.0948 ± 0.023 (24)	0.063 ± 0.033 (7.3)
**3**	0.18	0.486 ± 0.075 (7.4)	0.313 ± 0.186 (8.7)	0.149 ± 0.076 (20)	0.161 ± 0.040 (8.3)	0.0844 ± 0.0287 (27)	0.045 ± 0.016 (10)

## Supplementary Materials

Supplementary materials can be found at https://www.mdpi.com/1422-0067/20/12/3074/s1.

## Abbreviations

A2780	ovarian endometroid adenocarcinoma cell line
A-549	lung carcinoma cell line
AR	accumulation ratio
COX	cyclooxygenase
Cq	cycle quantification value
DMEM	Dulbecco’s Modified Eagle Medium
DMF	*N*,*N*-dimethylformamide
DNA	deoxyribonucleic acid
EDTA	2,2′,2″,2‴-(ethane-1,2-diyldinitrilo)tetraacetic acid or ethylenediaminetetraacetic acid
ESI-MS	electrospray ionization - mass spectrometry
FBS	fetal bovine serum
HCT 116	colorectal carcinoma cell line
HEPES	4-(2-hydroxyethyl)-1-piperazineethanesulfonic acid
HT-29	colon adenocarcinoma cell line
IC_50_	half-maximal inhibitory concentrations
ICP-MS	inductively coupled plasma - mass spectrometry
ICP-OES	inductively coupled plasma – optical emission spectroscopy
*k*′	RP-HPLC capacity factor
MPM	malignant pleural mesothelioma
MSTO-211H	biphasic malignant pleural mesothelioma cell line
NAG-1	NSAID activated gene 1
NMR	nuclear magnetic resonance
NSAIDs	non-steroidal anti-inflammatory drugs
PBS	phosphate buffered saline
PTFE	polytetrafluoroethylene
RNA	ribonucleic acid
RP-HPLC	reversed-phase high performance liquid chromatography
RPMI	Roswell Park Memorial Institute
RT-qPCR	quantitative reverse transcription polymerase chain reaction
SD	standard deviation
SEM	standard error means
SW480	colorectal adenocarcinoma
*t*_0_	column dead time
*t*_R_	retention time
UV	ultraviolet
ΔCq/ΔΔCq	cDNA level of expression

## References

[1] Oun R., Moussa Y.E., Wheate N.J. (2018). The side effects of platinum-based chemotherapy drugs: A review for chemists. Dalton Trans..

[2] Nakamura T., Miki T. (2010). Recent strategy for the management of advanced testicular cancer. Int. J. Urol..

[3] Morphy R., Kay C., Rankovic Z. (2004). From magic bullets to designed multiple ligands. Drug Discov. Today.

[4] Gabano E., Ravera M., Osella D. (2014). Pros and cons of bifunctional platinum(IV) antitumor prodrugs: Two are (not always) better than one. Dalton Trans..

[5] Apps M.G., Choi E.H.Y., Wheate N.J. (2015). The state-of-play and future of platinum drugs. Endoc. Relat. Cancer.

[6] Gibson D. (2016). Platinum(IV) anticancer prodrugs—Hypotheses and facts. Dalton Trans..

[7] Kenny R.G., Chuah S.W., Crawford A., Marmion C.J. (2017). Platinum(IV) Prodrugs—A Step Closer to Ehrlich’s Vision?. Eur. J. Inorg. Chem..

[8] Shi Y., Liu S.A., Kerwood D.J., Goodisman J., Dabrowiak J.C. (2012). Pt(IV) complexes as prodrugs for cisplatin. J. Inorg. Biochem..

[9] Wexselblatt E., Gibson D. (2012). What do we know about the reduction of Pt(IV) pro-drugs?. J. Inorg. Biochem..

[10] Johnstone T.C., Suntharalingam K., Lippard S.J. (2016). The Next Generation of Platinum Drugs: Targeted Pt(II) Agents, Nanoparticle Delivery, and Pt(IV) Prodrugs. Chem. Rev..

[11] Najjar A., Rajabi N., Karaman R. (2017). Recent Approaches to Platinum(IV) Prodrugs: A Variety of Strategies for Enhanced Delivery and Efficacy. Curr. Pharm. Design.

[12] Gibson D. (2019). Multi-action Pt(IV) anticancer agents; do we understand how they work?. J. Inorg. Biochem..

[13] Kenny R.G., Marmion C.J. (2019). Toward Multi-Targeted Platinum and Ruthenium Drugs—A New Paradigm in Cancer Drug Treatment Regimens?. Chem. Rev..

[14] Ravera M., Gabano E., McGlinchey M.J., Osella D. (2019). A view on multi-action Pt(IV) antitumor prodrugs. Inorg. Chim. Acta.

[15] Smith W.L., DeWitt D.L., Garavito R.M. (2000). Cyclooxygenases: Structural, cellular, and molecular biology. Annu. Rev. Biochem..

[16] Yu T.T., Lao X.Z., Zheng H. (2016). Influencing COX-2 Activity by COX Related Pathways in Inflammation and Cancer. Mini Rev. Med. Chem..

[17] Gurpinar E., Grizzle W.E., Piazza G.A. (2014). NSAIDs Inhibit Tumorigenesis, but How?. Clinical Cancer Res..

[18] Liggett J.L., Zhang X.B., Eling T.E., Baek S.J. (2014). Anti-tumor activity of non-steroidal anti-inflammatory drugs: Cyclooxygenase-independent targets. Cancer Lett..

[19] Cuzick J., Otto F., Baron J.A., Brown P.H., Burn J., Greenwald P., Jankowski J., La Vecchia C., Meyskens F., Senn H.J. (2009). Aspirin and non-steroidal anti-inflammatory drugs for cancer prevention: An international consensus statement. Lancet Oncol..

[20] Lin J., Hsiao P.W., Chiu T.H., Chao J.I. (2005). Combination of cyclooxygenase-2 inhibitors and oxaliplatin increases the growth inhibition and death in human colon cancer cells. Biochem. Pharmacol..

[21] Dannhardt G., Kiefer W. (2001). Cyclooxygenase inhibitors - current status and future prospects. Eur. J. Med. Chem..

[22] Thun M.J., Henley S.J., Patrono C. (2002). Nonsteroidal anti-inflammatory drugs as anticancer agents: Mechanistic, pharmacologic, and clinical issues. J. Natl. Cancer Inst..

[23] Patrignani P., Patrono C. (2015). Cyclooxygenase inhibitors: From pharmacology to clinical read-outs. Biochim. Biophys. Acta Mol. Cell Biol. Lipids.

[24] Pathak R.K., Marrache S., Choi J.H., Berding T.B., Dhar S. (2014). The Prodrug Platin-A: Simultaneous Release of Cisplatin and Aspirin. Angew. Chem. Int. Ed..

[25] Cheng Q.Q., Shi H.D., Wang H.X., Min Y.Z., Wang J., Liu Y.Z. (2014). The ligation of aspirin to cisplatin demonstrates significant synergistic effects on tumor cells. Chem. Commun..

[26] Cheng Q.Q., Shi H.D., Wang H.X., Wang J., Liu Y.Z. (2016). Asplatin enhances drug efficacy by altering the cellular response. Metallomics.

[27] Neumann W., Crews B.C., Marnett L.J., Hey-Hawkins E. (2014). Conjugates of Cisplatin and Cyclooxygenase Inhibitors as Potent Antitumor Agents Overcoming Cisplatin Resistance. ChemMedChem.

[28] Neumann W., Crews B.C., Sarosi M.B., Daniel C.M., Ghebreselasie K., Scholz M.S., Marnett L.J., Hey-Hawkins E. (2015). Conjugation of Cisplatin Analogues and Cyclooxygenase Inhibitors to Overcome Cisplatin Resistance. ChemMedChem.

[29] Curci A., Denora N., Iacobazzi R.M., Ditaranto N., Hoeschele J.D., Margiotta N., Natile G. (2018). Synthesis, characterization, and in vitro cytotoxicity of a Kiteplatin-Ibuprofen Pt(IV) prodrug. Inorg. Chim. Acta.

[30] Tan J.J., Li C., Wang Q., Li S.Y., Chen S.Z., Zhang J.C., Wang P.C., Ren L., Liang X.J. (2018). A Carrier-Free Nanostructure Based on Platinum(IV) Prodrug Enhances Cellular Uptake and Cytotoxicity. Mol. Pharm..

[31] Qin X.D., Xu G., Chen F.H., Fang L., Gou S.H. (2017). Novel platinum(IV) complexes conjugated with a wogonin derivative as multi-targeted anticancer agents. Bioorg. Med. Chem..

[32] Chen F.H., Qin X.D., Xu G., Gou S.H., Jin X.F. (2017). Reversal of cisplatin resistance in human gastric cancer cells by a wogonin-conjugated Pt(IV) prodrug via attenuating Casein Kinase 2-mediated Nuclear Factor-kappa B pathways. Biochem.Pharmacol..

[33] Maiti S., Paira P. (2018). Biotin conjugated organic molecules and proteins for cancer therapy: A review. Eur. J. Med. Chem..

[34] Hu W.W., Fang L., Hua W.Y., Gou S.H. (2017). Biotin-Pt(IV)-indomethacin hybrid: A targeting anticancer prodrug providing enhanced cancer cellular uptake and reversing cisplatin resistance. J. Inorg. Biochem..

[35] Tolan D.A., Abdel-Monem Y.K., El-Nagar M.A. (2019). Anti-tumor platinum(IV) complexes bearing the anti-inflammatory drug naproxen in the axial position. Appl. Organ. Chem..

[36] Cryer B., Feldman M. (1998). Cyclooxygenase-1 and cyclooxygenase-2 selectivity of widely used nonsteroidal anti-inflammatory drugs. Am. J. Med..

[37] Wilson J.J., Lippard S.J. (2014). Synthetic Methods for the Preparation of Platinum Anticancer Complexes. Chem. Rev..

[38] Ravera M., Gabano E., Zanellato I., Fregonese F., Pelosi G., Platts J.A., Osella D. (2016). Antiproliferative activity of a series of cisplatin-based Pt(IV)-acetylamido/carboxylato prodrugs. Dalton Trans..

[39] Gabano E., Gama S., Mendes F., Fregonese F., Paulo A., Ravera M. (2015). Application of microwave-assisted heating to the synthesis of Pt(II) complexes. Inorg. Chim. Acta.

[40] Petruzzella E., Chirosca C.V., Heidenga C.S., Hoeschele J.D. (2015). Microwave-assisted synthesis of the anticancer drug cisplatin, *cis*-Pt(NH_3_)_2_Cl_2_. Dalton Trans..

[41] Gabano E., Ravera M., Trivero F., Tinello S., Gallina A., Zanellato I., Gariboldi M.B., Monti E., Osella D. (2018). The cisplatin-based Pt(IV)-diclorofibrato multi-action anticancer prodrug exhibits excellent performances also under hypoxic conditions. Dalton Trans..

[42] Gabano E., Ravera M., Perin E., Zanellato I., Rangone B., McGlinchey M.J., Osella D. (2019). Synthesis and characterization of cyclohexane-1*R*,2R-diamine-based Pt(IV) dicarboxylato anticancer prodrugs: Their selective activity against human colon cancer cell lines. Dalton Trans..

[43] Liu X., Testa B., Fahr A. (2011). Lipophilicity and Its Relationship with Passive Drug Permeation. Pharm. Res..

[44] Lin X.L., Hefesha H., Scriba G., Fahr A. (2008). Retention behavior of neutral and positively and negatively charged solutes on an immobilized-artificial-membrane (IAM) stationary phase. Helvet. Chim. Acta.

[45] Nemirovski A., Kasherman Y., Tzaraf Y., Gibson D. (2007). Reduction of *cis,trans,cis*-PtCl_2_(OCOCH_3_)_2_(NH_3_)_2_ by aqueous extracts of cancer cells. J. Med. Chem..

[46] Gabano E., Ravera M., Zanellato I., Tinello S., Gallina A., Rangone B., Gandin V., Marzano C., Bottone M.G., Osella D. (2017). An unsymmetric cisplatin-based Pt(IV) derivative containing 2-(2-propynyl) octanoate: A very efficient multi-action antitumor prodrug candidate. Dalton Trans..

[47] Ronconi L., Sadler P.J. (2008). Applications of heteronuclear NMR spectroscopy in biological and medicinal inorganic chemistry. Coord. Chem. Rev..

[48] Davies M.S., Hall M.D., Berners-Price S.J., Hambley T.W. (2008). ^1^H, ^15^N heteronuclear single quantum coherence NMR study of the mechanism of aquation of platinum(IV) ammine complexes. Inorg. Chem..

[49] Catalano A., Graciotti L., Rinaldi L., Raffaelli G., Rodilosso S., Betta P., Gianni W., Amoroso S., Procopio A. (2004). Reclinical evaluation of the nonsteroidal anti-inflammatory agent celecoxib on malignant mesothelioma chemoprevention. Int. J. Cancer.

[50] Livak K.J., Schmittgen T.D. (2001). Analysis of relative gene expression data using real-time quantitative PCR and the 2^−ΔΔC^ method. Methods.

[51] Nolan T., Hands R.E., Bustin S.A. (2006). Quantification of mRNA using real-time RT-PCR. Nat. Protocols.

[52] Chou T.C., Talalay P. (1984). Quantitative analysis of dose-effect relationships: The combined effects of multiple drugs or enzyme inhibitors. Adv. Enzyme Regul..

[53] Chou T.-C. (2006). Theoretical basis, experimental design, and computerized simulation of synergism and antagonism in drug combination studies. Pharmacol. Rev..

[54] Chou T.C. (2010). Drug Combination Studies and Their Synergy Quantification Using the Chou-Talalay Method. Cancer Res..

[55] Lindauer E., Holler E. (1996). Cellular distribution and cellular reactivity of platinum(II) complexes. Biochem. Pharmacol.y.

[56] Alessio M., Zanellato I., Bonarrigo I., Gabano E., Ravera M., Osella D. (2013). Antiproliferative activity of Pt(IV)-*bis*(carboxylato) conjugates on malignant pleural mesothelioma cells. J. Inorg. Biochem..

[57] Raveendran R., Braude J.P., Wexselblatt E., Novohradsky V., Stuchlikova O., Brabec V., Gandin V., Gibson D. (2016). Pt(IV) derivatives of cisplatin and oxaliplatin with phenylbutyrate axial ligands are potent cytotoxic agents that act by several mechanisms of action. Chem. Sci..

[58] Singh R., Letai A., Sarosiek K. (2019). Regulation of apoptosis in health and disease: The balancing act of BCL-2 family proteins. Nat. Rev. Mol. Cell Biol..

[59] Baek S.J., Kim J.S., Moore S.M., Lee S.H., Martinez J., Eling T.E. (2005). Cyclooxygenase inhibitors induce the expression of the tumor suppressor gene EGR-1, which results in the up-regulation of NAG-1, an antitumorigenic protein. Mol. Pharmacol..

[60] Baek S.J., Kim K.S., Nixon J.B., Wilson L.C., Eling T.E. (2001). Cyclooxygenase inhibitors regulate the expression of a TGF-beta superfamily member that has proapoptotic and antitumorigenic activities. Mol. Pharmacol..

[61] Iczkowski K.A., Pantazis C.G. (2003). Overexpression of NSAID-activated gene product in prostate cancer. Int. J. Surg. Pathol..

[62] Iguchi G., Chrysovergis K., Lee S.H., Baek S.J., Langenbach R., Eling T.E. (2009). A reciprocal relationship exists between non-steroidal anti-inflammatory drug-activated gene-1 (NAG-1) and cyclooxygenase-2. Cancer Lett..

[63] Baek S.J., Wilson L.C., Lee C.H., Eling T.E. (2002). Dual function of nonsteroidal anti-inflammatory drugs (NSAIDs): Inhibition of cyclooxygenase and induction of NSAID-activated gene. J. Pharmacol. Exp. Therap..

[64] Wilson L.C., Baek S.J., Call A., Eling T.E. (2003). Nonsteroidal anti-inflammatory drug-activated gene (NAG-1) is induced by genistein through the expression of p53 in colorectal cancer cells. Int. J. Cancer.

[65] Eling T.E., Baek S.J., Shim M., Lee C.H. (2006). NSAID activated gene (NAG-1), a modulator of tumorigenesis. J. Biochem. Mol. Biol..

[66] Parfenova H., Parfenov V.N., Shlopov B.V., Levine V., Falkos S., Pourcyrous M., Leffler C.W. (2001). Dynamics of nuclear localization sites for COX-2 in vascular endothelial cells. Am. J. Physiol. Cell Physiol..

[67] Yamashita M., Tsuji S., Nishiyama A., Myrvik Q.N., Henriksen R.A., Shibata Y. (2007). Differential subcellular localization of COX-2 in macrophages phagocytosing heat-killed Mycobacterium bovis BCG. Am. J. Physiol. Cell Physiol..

[68] Ravera M., Gabano E., Tinello S., Zanellato I., Osella D. (2017). May glutamine addiction drive the delivery of antitumor cisplatin-based Pt(IV) prodrugs?. J. Inorg. Biochem..

[69] Ermondi G., Caron G., Ravera M., Gabano E., Bianco S., Platts J.A., Osella D. (2013). Molecular interaction fields vs. quantum-mechanical-based descriptors in the modelling of lipophilicity of platinum(iv) complexes. Dalton Trans..

[70] Platts J.A., Ermondi G., Caron G., Ravera M., Gabano E., Gaviglio L., Pelosi G., Osella D. (2011). Molecular and statistical modeling of reduction peak potential and lipophilicity of platinum(IV) complexes. J. Biol. Inorg. Chem..

[71] Ravera M., Gabano E., Bianco S., Ermondi G., Caron G., Vallaro M., Pelosi G., Zanellato I., Bonarrigo I., Cassino C. (2015). Host-guest inclusion systems of Pt(IV)-bis(benzoato) anticancer drug candidates and cyclodextrins. Inorg. Chim. Acta.

[72] Ravera M., Gabano E., Zanellato I., Ilaria B., Alessio M., Arnesano F., Galliani A., Natile G., Osella D. (2015). Cellular trafficking, accumulation and DNA platination of a series of cisplatin-based dicarboxylato Pt(IV) prodrugs. J. Inorg. Biochem..

