# Supplementary material for: Antiproliferative Activity of Pt(IV) Conjugates Containing the Non-Steroidal Anti-Inflammatory Drugs (NSAIDs) Ketoprofen and Naproxen
Source: Int J Mol Sci. 2019 Jun 24;20(12):3074. doi: 10.3390/ijms20123074 (PMC6627341; doi:10.3390/ijms20123074)
Supplement: Supplementary file 1 [file ijms-20-03074-s001.pdf]

Antiproliferative activity of Pt(IV) conjugates containing the Non-Steroidal Anti-Inflammatory Drugs (NSAIDs) ketoprofen and naproxen

Mauro Ravera, Ilaria Zanellato, Elisabetta Gabano, Beatrice Rangone, Marco Coppola, Elena Perin, Domenico Osella*

Dipartimento di Scienze e Innovazione Tecnologica, Università del Piemonte Orientale, Viale Michel 11, 15121 Alessandria (Italy)

SUPPLEMENTARY MATERIAL

CONTENT:

Figure S1. Sketch of the complexes under investigation.

Figure S2-Figure S3. NMR characterisation of (*RS*)-2-[3-(benzoyl)phenyl]propanoyl chloride

Figure S4-Figure S5. NMR characterisation of 2-(6-metoxynaphtalen-2-yl)propanoyl chloride

Figure S6-Figure S9. MS and NMR characterisation of complex **2**

Figure S10-Figure S13. MS and NMR characterisation of complex **3**

Figure S14-Figure S17. Residual viability (resazurin reduction assay) data of HCT 116 and HT-29 cells after treatment with cisplatin, NSAIDs and a cisplatin : NSAIDs 1 : 500 mixture. These data were used to obtain the Combination Index (CI) value with the method of Chou and Talalay.

Figure S18-Figure S19. Representative pictures of HCT 116 and A-549 cells 24 h after a treatment with **2** and **3**.

Table S1. Genes analyzed by means of Quantitative Reverse Transcription PCR (RT-qPCR).

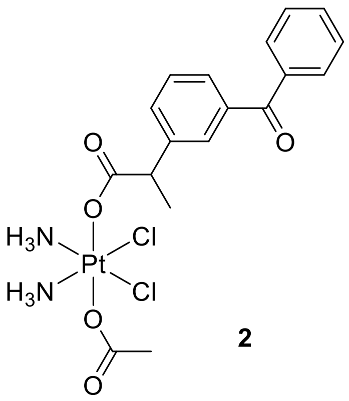

2

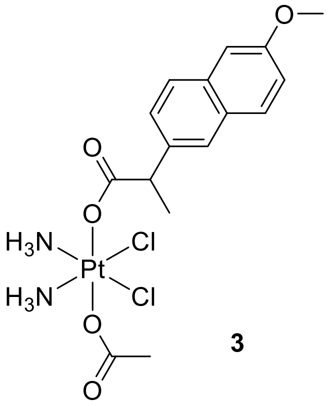

3

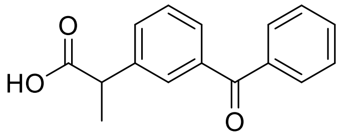

ketoprofen

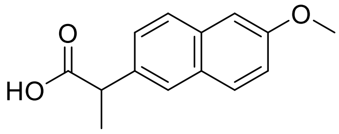

naproxen

Figure S1. Sketch of the complexes under investigation.

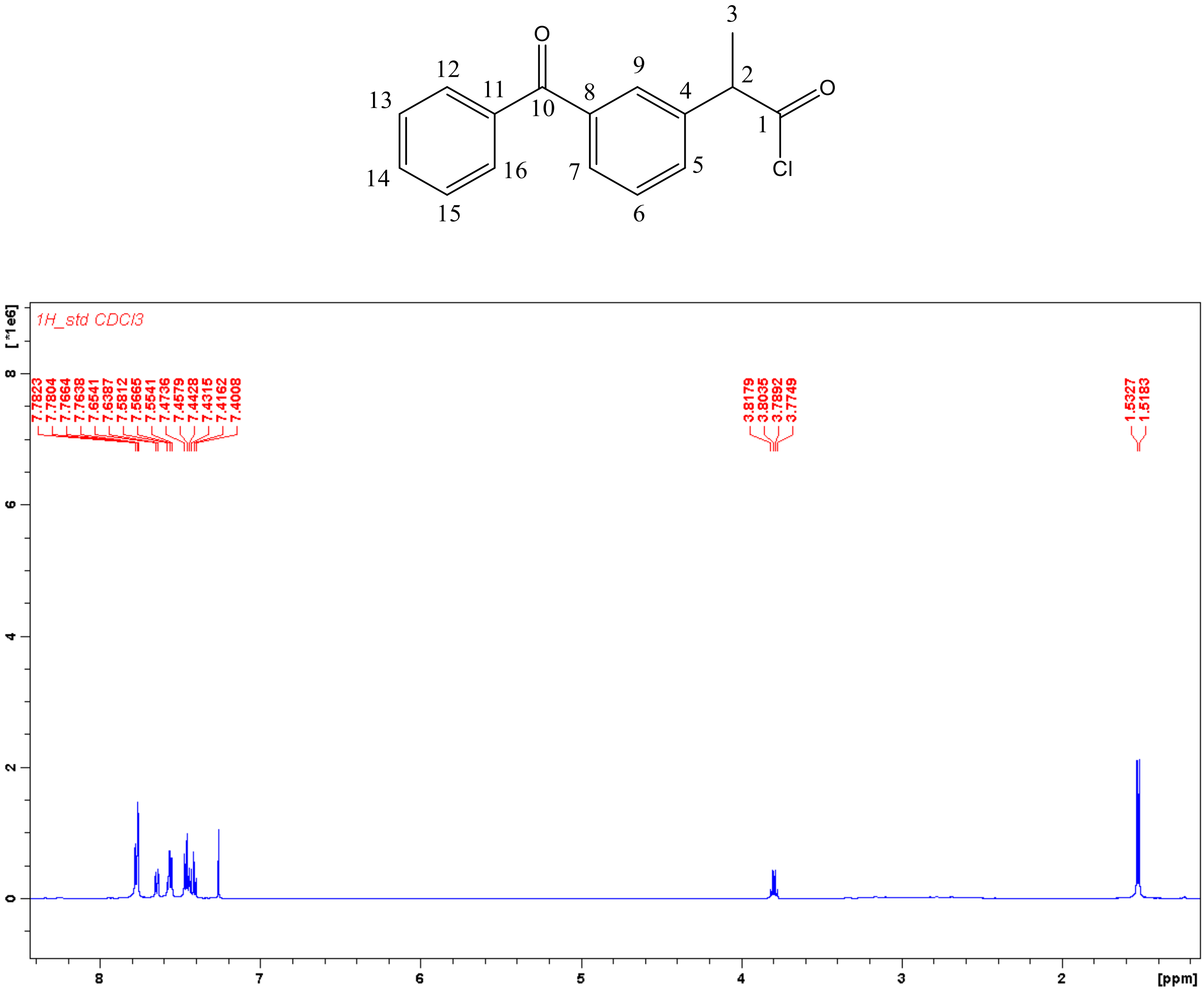

Figure S2. ^{1}H-NMR spectrum of (*RS*)-2-[3-(benzoyl)phenyl]propanoyl chloride in $CDCl_3$.

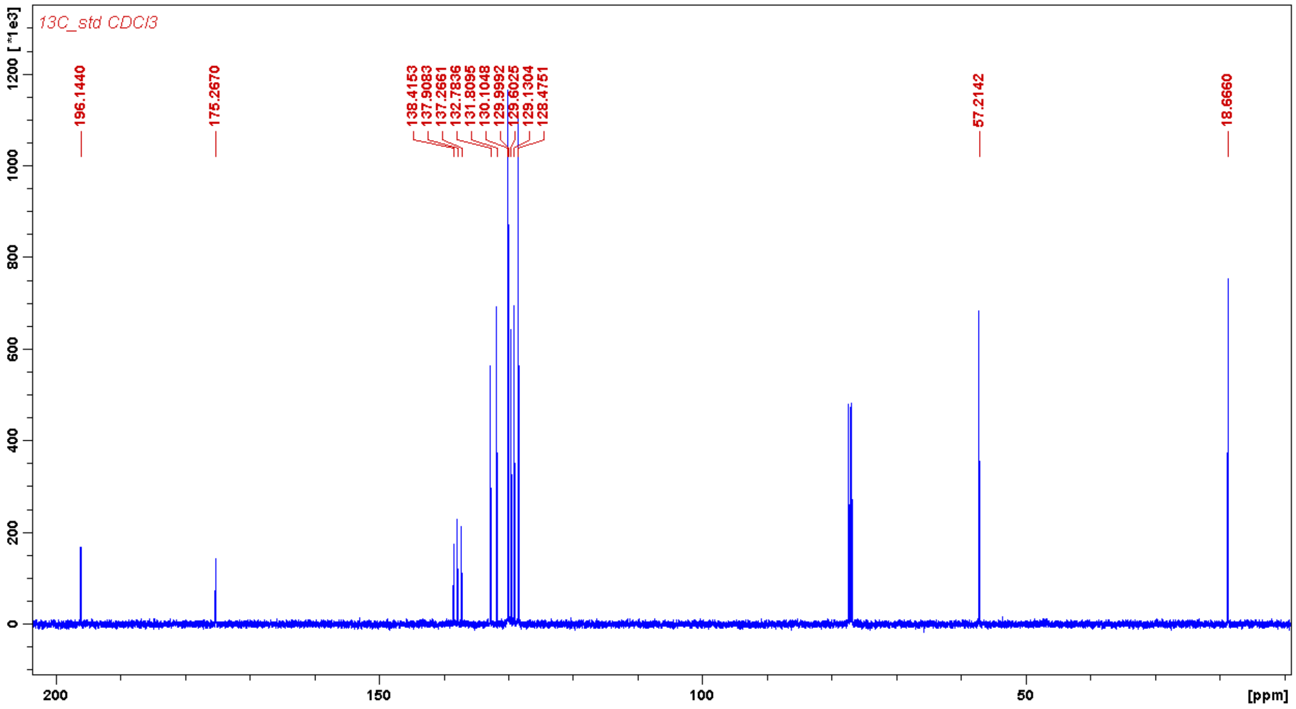

Figure S3. ^{13}C-NMR spectrum of (*RS*)-2-[3-(benzoyl)phenyl]propanoyl chloride in $CDCl_3$.

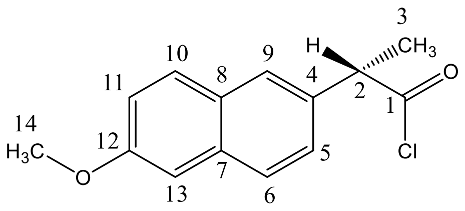

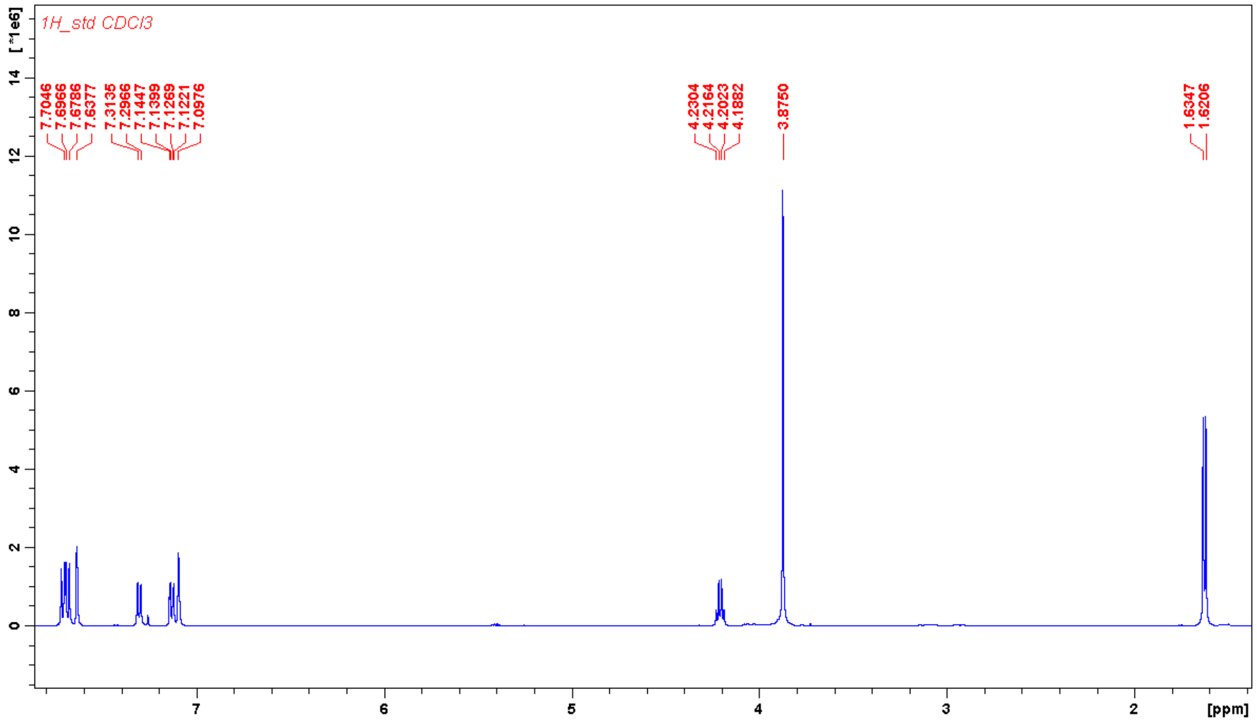

Figure S4. ^{1}H -NMR spectrum of 2-(6-metoxynaphtalen-2-yl)propanoyl chloride in $CDCl_3$.

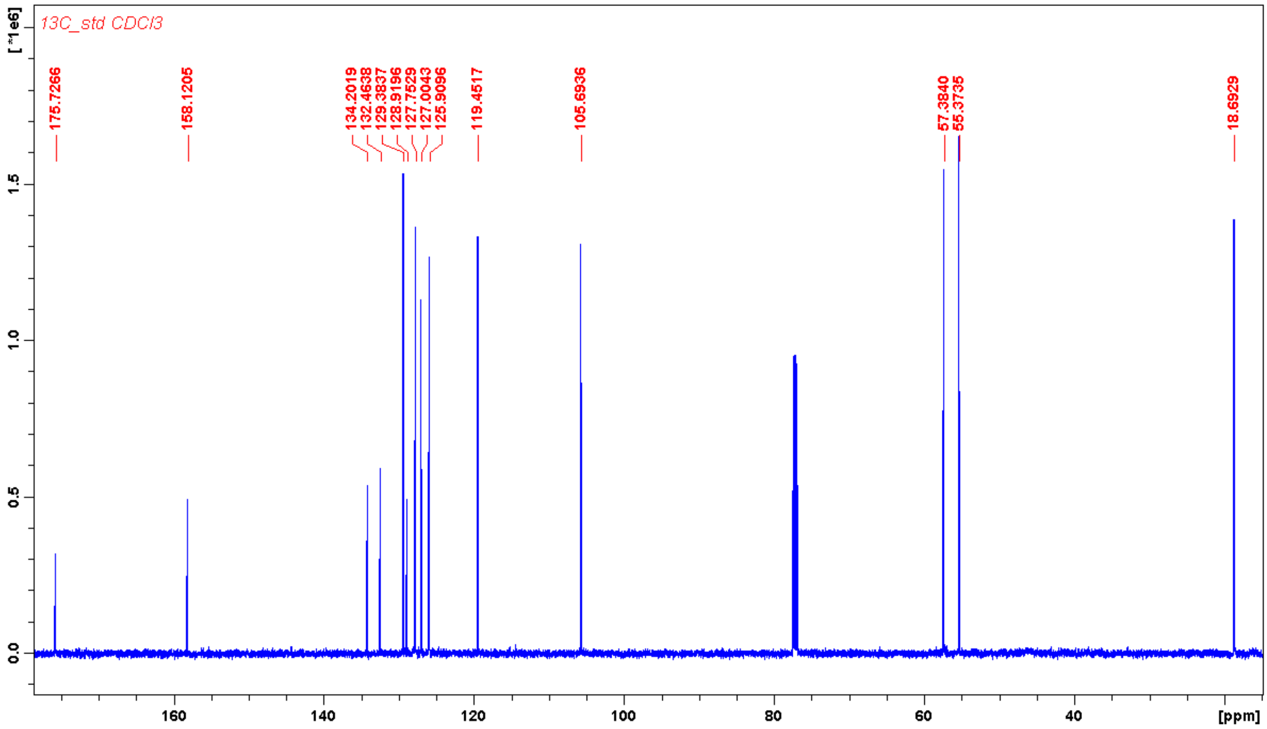

Figure S5. ^{13}C -NMR spectrum of 2-(6-metoxynaphtalen-2-yl)propanoyl chloride in $CDCl_3$.

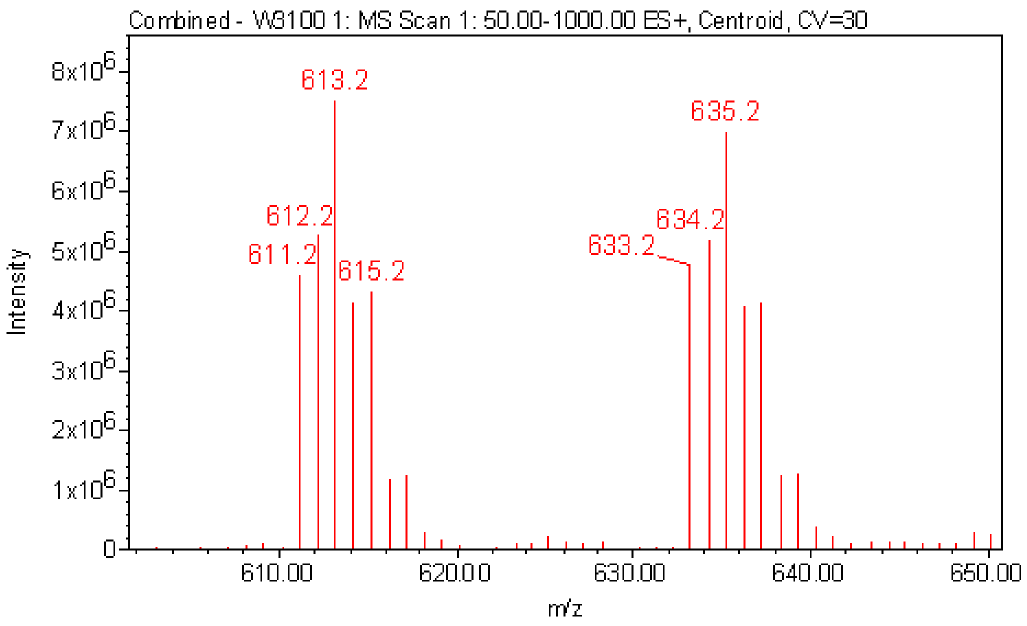

Figure S6. ESI-MS spectrum of complex **2**

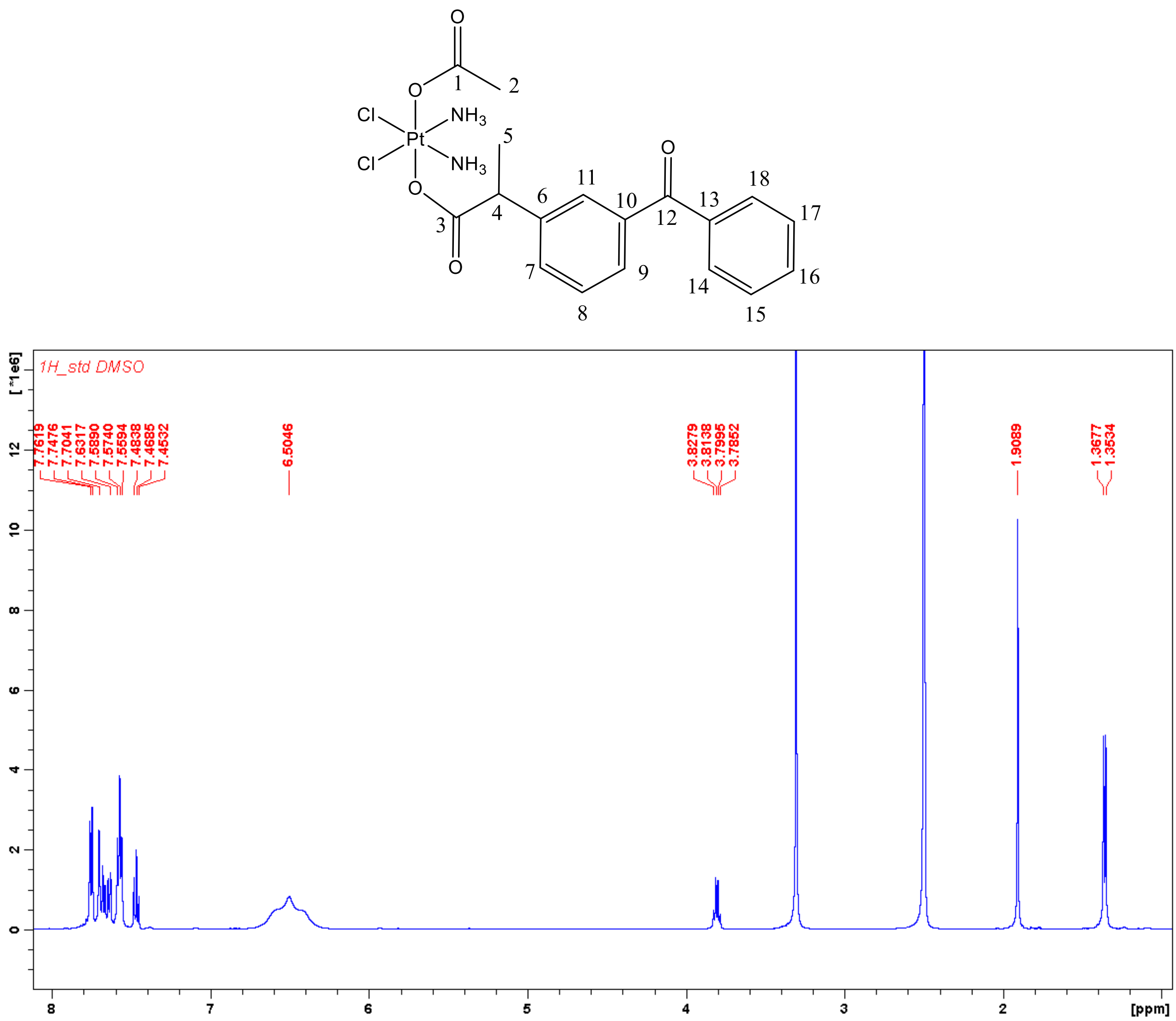

Figure S7. ^{1}H-NMR spectrum of complex **2** in DMSO-d_6.

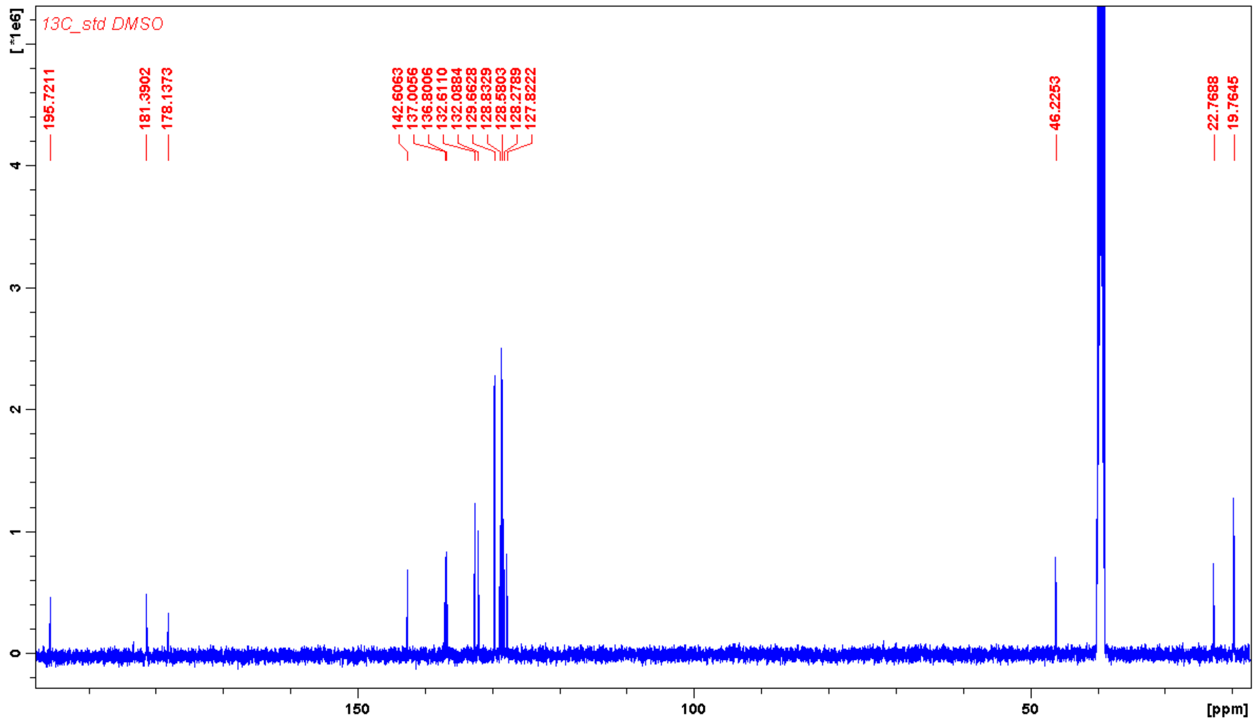

Figure S8. ^{13}C-NMR spectrum of complex **2** in DMSO-d_6

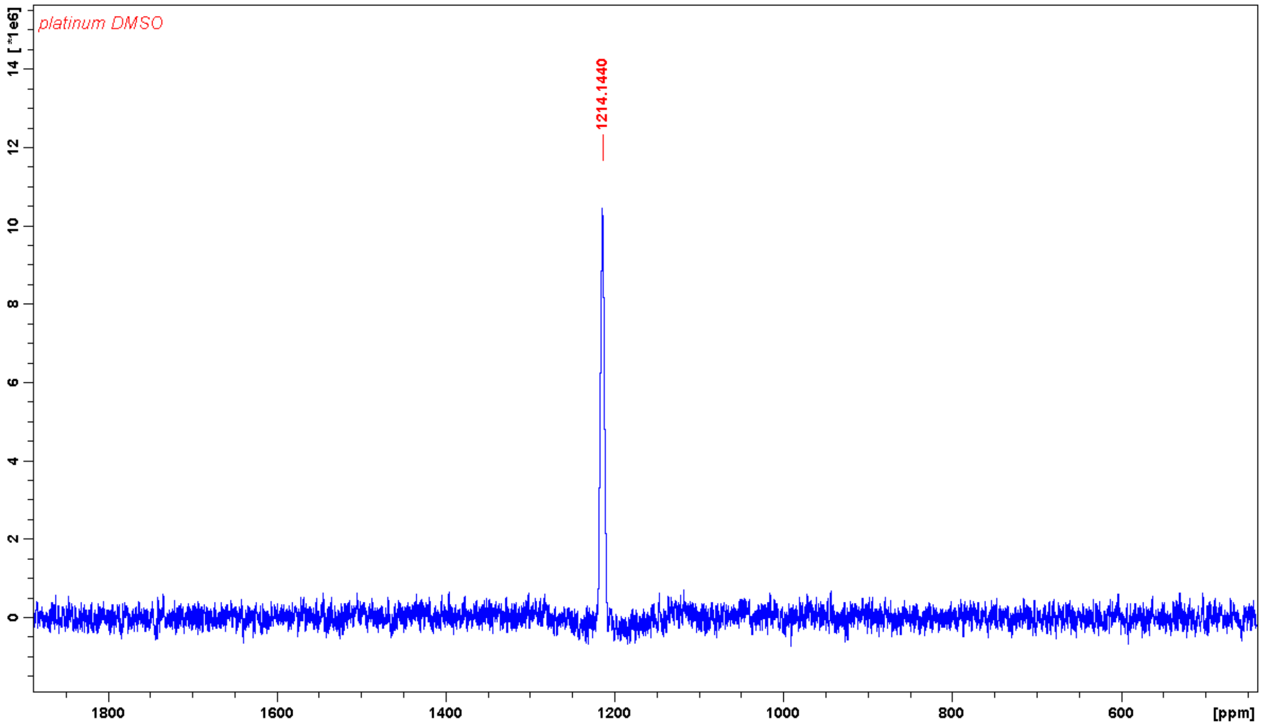

Figure S9. ^{195}Pt-NMR spectrum of complex **2** in DMSO-d_6

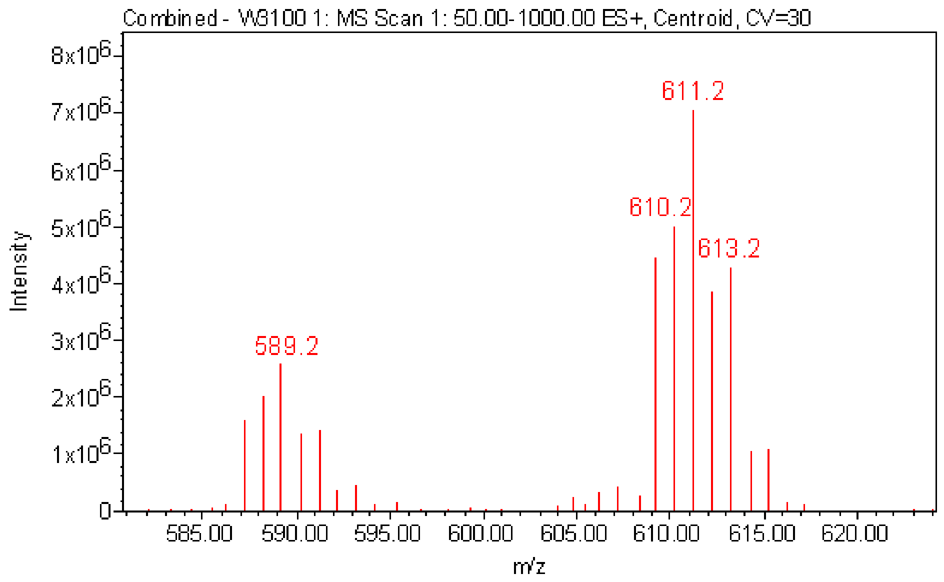

Figure S10. ESI-MS spectrum of complex **3**

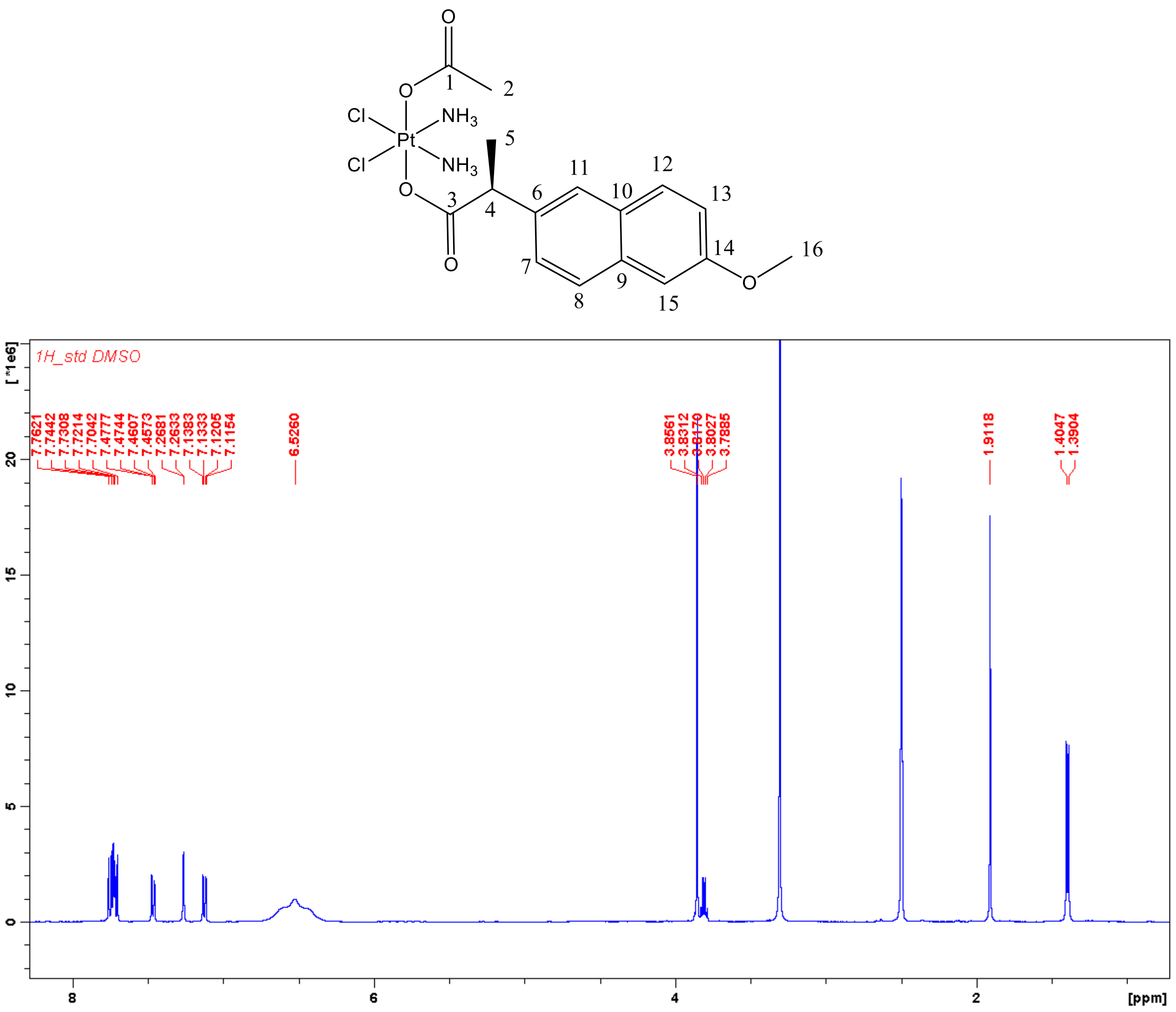

Figure S11. ^{1}H-NMR spectrum of complex **3** in DMSO-d_6.

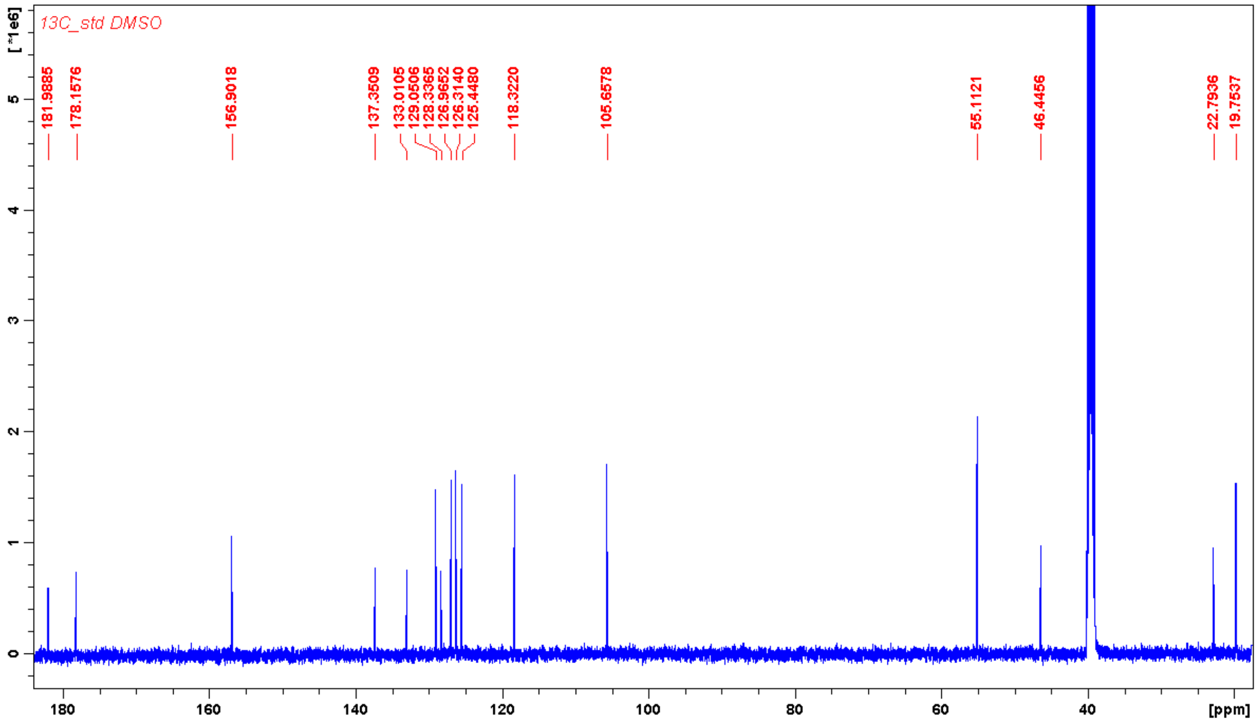

Figure S12. ^{13}C-NMR spectrum of complex **3** in DMSO-d_6.

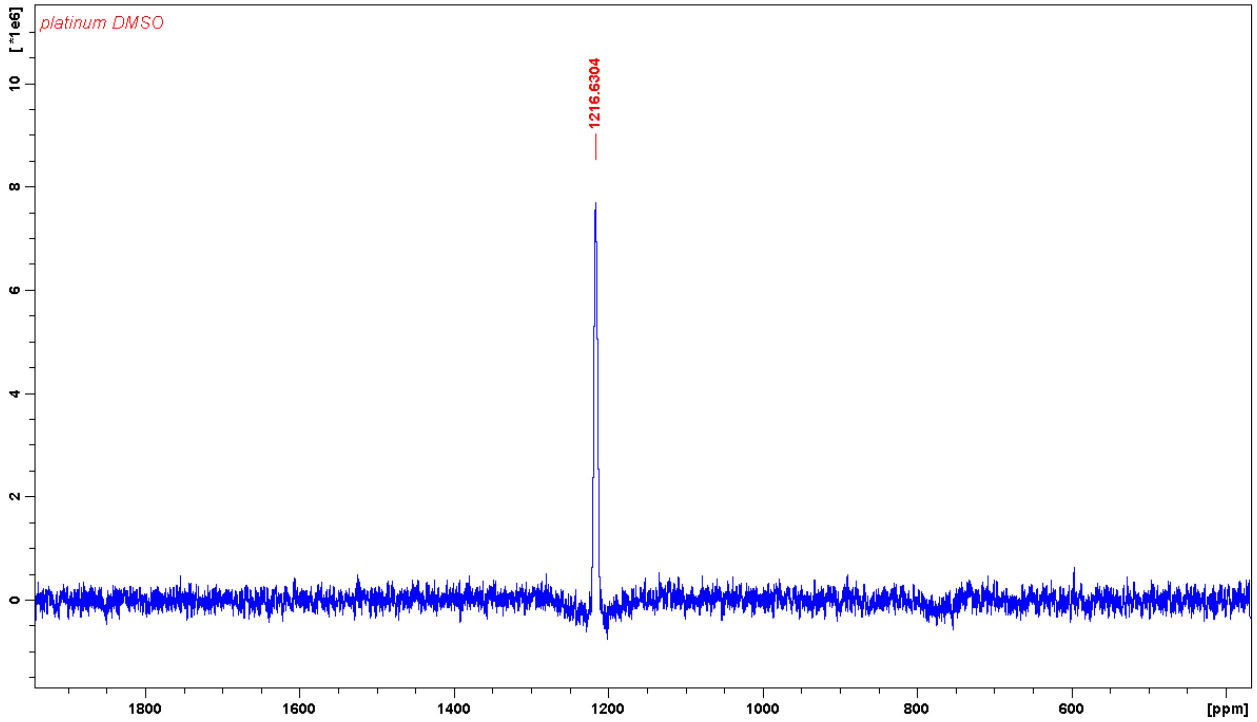

Figure S13. ^{195}Pt-NMR spectrum of complex **3** in DMSO-d_6.

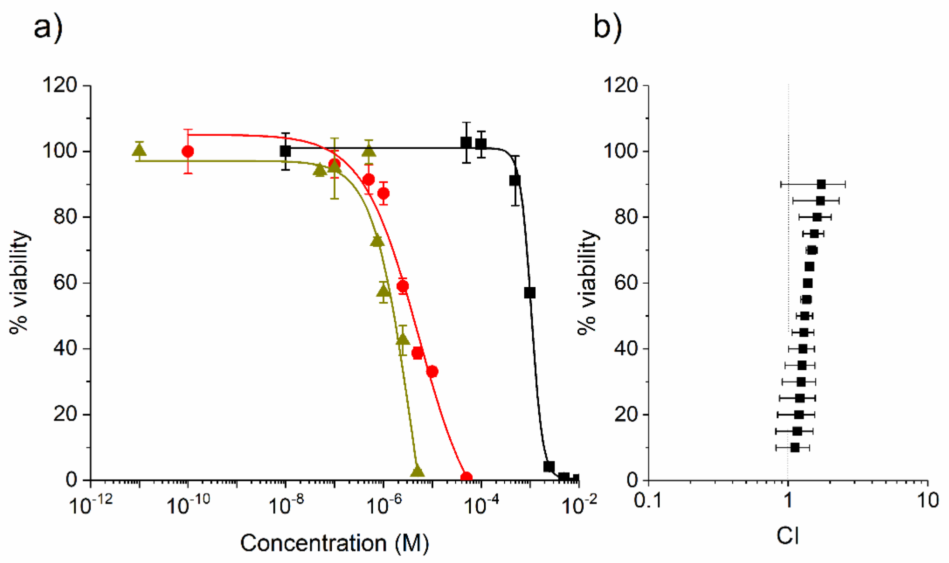

Figure S14. HCT116 cells were treated for 72 h with cisplatin (red dots), ketoprofen (black squares) or a cisplatin : ketoprofen mixture (in a fixed 1:500 ratio, according to their respective IC_{50} values, green triangles). a) Residual viability was assessed by means of the resazurin reduction assay and data were fitted with a four-parameter function. b) Residual viability data of the 1:500 mixture were compared to obtain the Combination Index (CI) value with the method of Chou and Talalay (equation for non-mutually exclusive drugs).

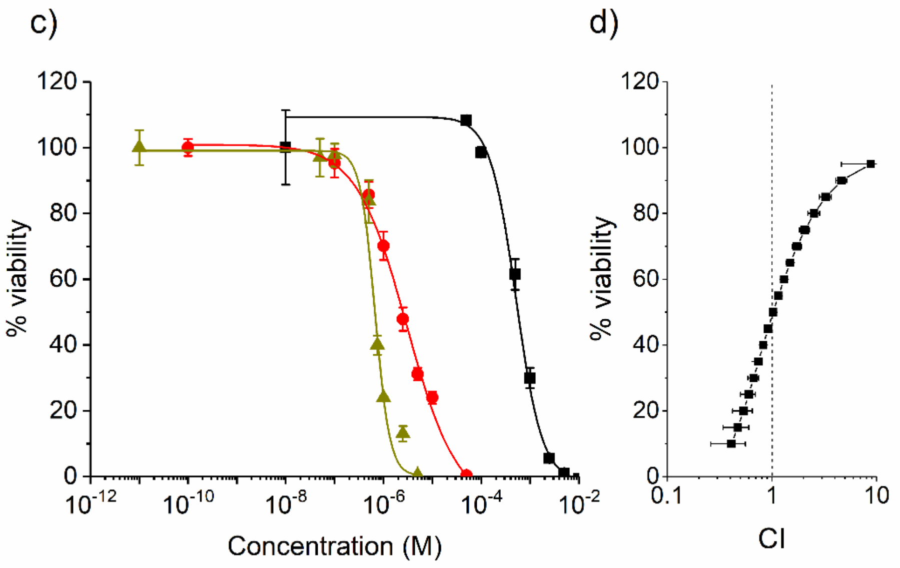

Figure S15. HCT116 cells were treated for 72 h with cisplatin (red dots), naproxen (black squares) or a cisplatin : naproxen mixture (in a fixed 1:500 ratio, according to their respective IC_{50} values, green triangles). c) Residual viability was assessed by means of the resazurin reduction assay and data were fitted with a four-parameter function. d) Residual viability data of the 1:500 mixture were compared to obtain the Combination Index (CI) value with the method of Chou and Talalay (equation for non-mutually exclusive drugs).

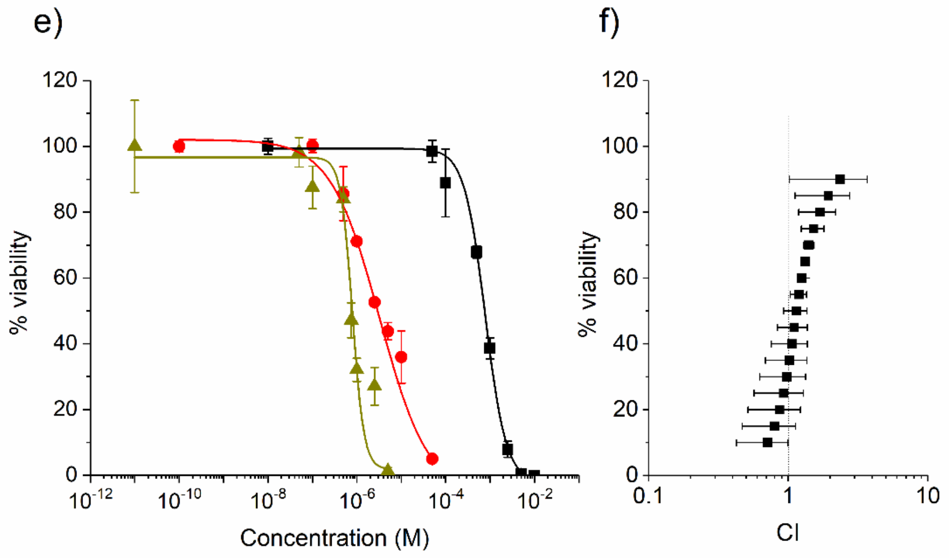

Figure S16. HT-29 cells were treated for 72 h with cisplatin (red dots), ketoprofen (black squares) or a cisplatin : ketoprofen mixture (in a fixed 1:500 ratio, according to their respective IC_{50} values, green triangles). e) Residual viability was assessed by means of the resazurin reduction assay and data were fitted with a four-parameter function. f) Residual viability data of the 1:500 mixture were compared to obtain the Combination Index (CI) value with the method of Chou and Talalay (equation for non-mutually exclusive drugs).

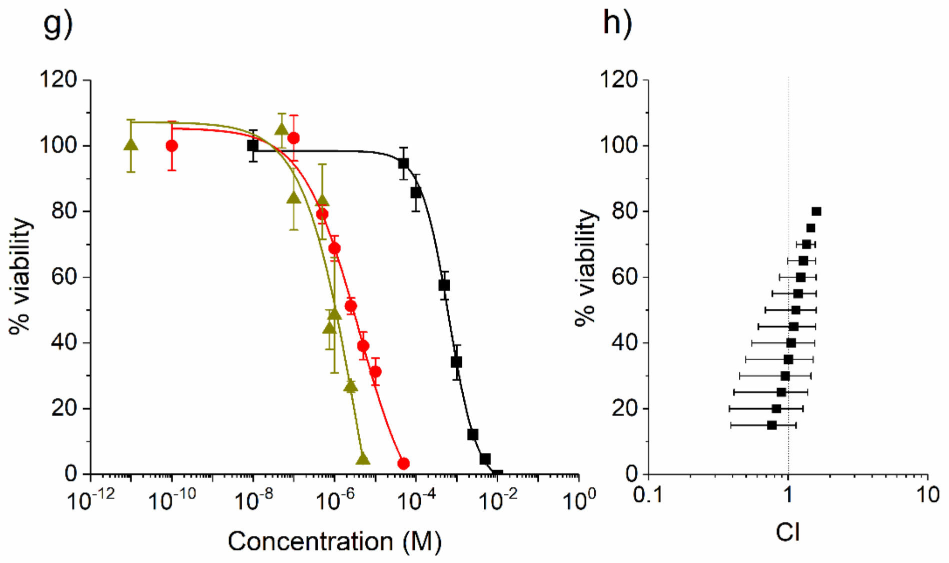

Figure S17. HT-29 cells were treated for 72 h with cisplatin (red dots), naproxen (black squares) or a cisplatin : naproxen mixture (in a fixed 1:500 ratio, according to their respective IC_{50} values, green triangles). g) Residual viability was assessed by means of the resazurin reduction assay and data were fitted with a four-parameter function. h) Residual viability data of the 1:500 mixture were compared to obtain the Combination Index (CI) value with the method of Chou and Talalay (equation for non-mutually exclusive drugs).

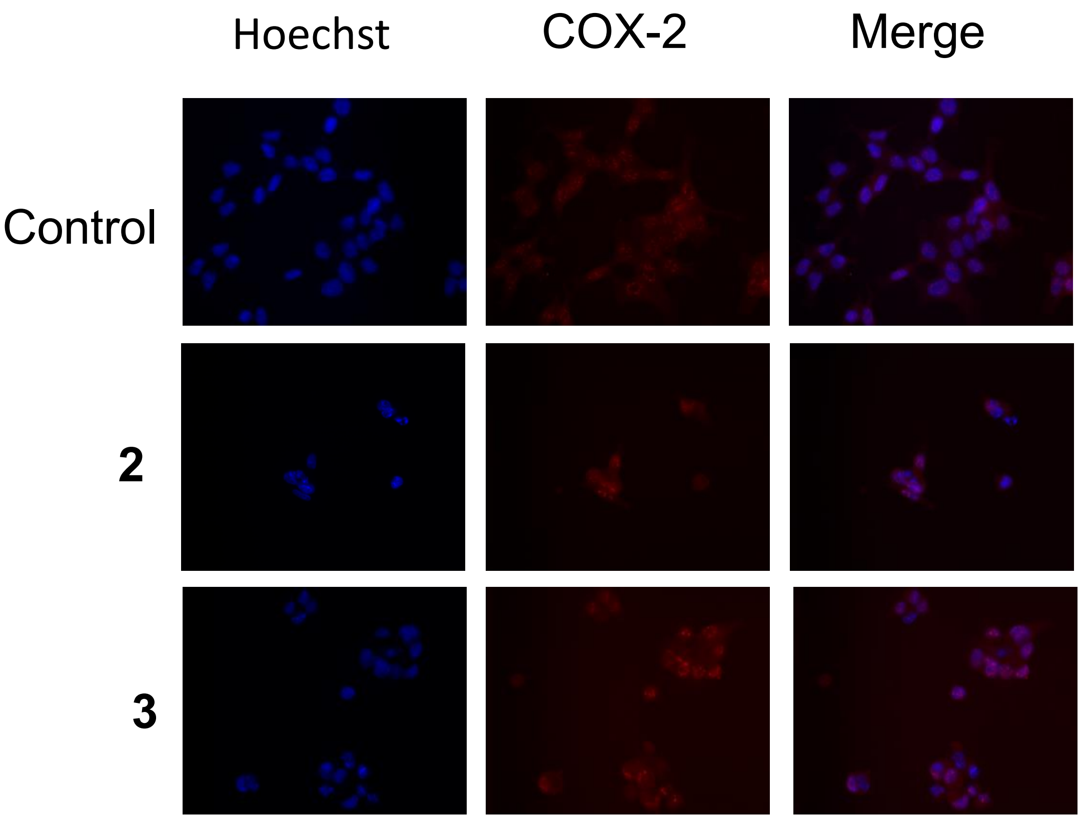

Figure S18. Representative pictures of HCT 116 cells 24 h after a 3 μM treatment with **2** and **3** (from top to bottom: control, **2**, and **3**). Nuclei were stained with Hoechst 33258 (left), COX-2 was revealed with an Alexa 594-conjugated secondary antibody (middle), and images were merged (right). Pictures taken by means of Olympus BX51 microscope at 40× magnification. (For the description of the experiments see note after Figure S19.)

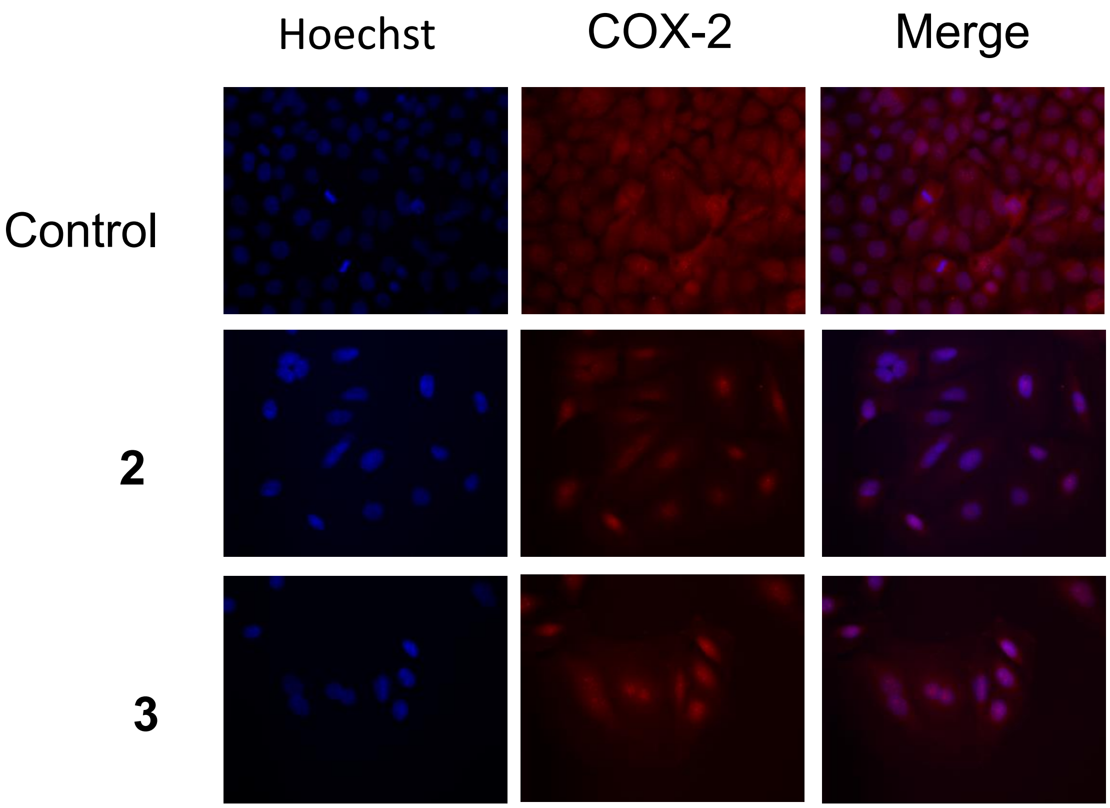

Figure S19. Representative pictures of A-549 cells 24 h after a 5 μM treatment with **2** and **3** (from top to bottom: control, **2**, and **3**). Nuclei were stained with Hoechst 33258 (left), COX-2 was revealed with an Alexa 594-conjugated secondary antibody (middle), and images were merged (right). Pictures taken by means of Olympus BX51 microscope at 40 × magnification.

Immunochemical analysis. HCT 116 and A-549 cells (5×10^5) were seeded on coverslips and allowed to grow for 24 h. The cells were treated with equitoxic concentrations of the compounds under investigation (*i.e.*, HCT 116: 3 μM for both complexes **2** and **3**; A-549: 5 μM for both complexes **2** and **3**. After 24 h, the samples were fixed with 4% formalin and post-fixed with 70% ethanol at -20 ℃ for at least 24 h. After rehydration in PBS for 10 minutes, the cells were incubated with a primary antibody against COX-2 (Santa Cruz Biotechnology), at 1:200 dilution, for 60 min. Then, the coverslips were washed three times with PBS and incubated with Alexa 594-conjugated anti-goat secondary antibody (Molecular Probes) at 1:200 dilution. All the incubations were performed in the dark at room temperature. Finally, the sections were counterstained for DNA with 0.1 μg mL^{-1} Hoechst 33258 (Sigma-Aldrich, Milano, Italy) for 10 minutes, washed with PBS, and mounted in a drop of Mowiol (Calbiochem, Inalco, Italy) for fluorescence microscopy analysis. An Olympus BX51 microscope equipped with a 100-W mercury lamp was used under the following conditions:

- for Hoechst 33258: 330–385 nm excitation filter, 400 nm dichroic mirror, and 420 nm barrier filter
- for Alexa 594: 540 nm excitation filter, 580 nm dichroic mirror, and 620 nm barrier filter.

Images were recorded with an Olympus MagniFire camera system and processed with the Olympus Cell F software.

Table S1. Genes analyzed by means of Quantitative Reverse Transcription PCR (RT-qPCR). The NCBI accession number is reported along with the 5'-3' sequence of the forward and reverse primer and the expected product length.

Gene	Accession no.	Forward	Reverse	Product lenght (bp)
**COX-2**	M90100.1	CCCTGAGCATCTACGGTTTG	CATCGCATACTCTGTTGTGTTC	107
**GAPDH**	NG_007073.2	ATCCCTGAGCTGAACGGGAA	GGCAGGTTTTTCTAGACGGC	99
**HPRT1**	NM_000194.2	TTGCTTTCCTTGGTCAGGCA	ATCCAACACTTCGTGGGGTC	85
**RNA18SN1**	NR_145820.1	CGTCTGCCCTATCAACTTTCG	TGCCTTCCTTGGATGTGGTAG	124
**BAX**	NM_001291428.1	GACCATCTTTGTGGCGGGAG	GAGGAAAAACACAGTCCAAGGC	94
**BAD**	NM_004322.3	GAGACCTGTGCGCCGTCA	AGGACCTCAGTCTCCCCTCAG	74
**Bcl2a**	NM_000633.2	CTTTGAGTTCGGTGGGGTCA	GGGCCGTACAGTTCCACAAA	162
**NAG-1/GDF15**	NM_004864.3	TTGCGGAAACGCTACGAGG	GCACTTCTGGCGTGAGTATCC	115

↑↓
